# Beyond phosphate uptake: PT4/PT11 as key functional components linking phosphate acquisition, nutrient exchange, and arbuscule maintenance in arbuscular mycorrhizal symbiosis

**DOI:** 10.3389/fpls.2026.1966117

**Published:** 2026-09-28

**Authors:** Xueying Yi, Ziming Ma

**Affiliations:** 1 Institute of Biotechnology, Chongqing Academy of Agricultural Sciences, Chongqing, China; 2 Root Biology and Symbiosis, Max-Planck-Institute of Molecular Plant Physiology, Potsdam, Germany; 3 Jilin Provincial Engineering Laboratory of Plant Genetic Improvement, College of Plant Science, Jilin University, Changchun, China

**Keywords:** arbuscule maintenance, common symbiosis signaling pathway, nutrient exchange, phosphate transporter PT4/PT11, transcriptional regulation

## Abstract

Phosphorus is an essential macronutrient for plant growth and development, yet its availability in many soils is limited by strong chemical fixation and low mobility. Arbuscular mycorrhizal (AM) symbiosis provides an important biological strategy for improving plant phosphate acquisition by establishing specialized arbuscules within root cortical cells, where reciprocal nutrient exchange occurs across the periarbuscular membrane (PAM). Among the phosphate transporter families involved in this process, the mycorrhiza-specific PHT1 transporters PT4 in *Medicago truncatula* and PT11 in *Oryza sativa* are major components of the mycorrhizal phosphate uptake pathway and are required for efficient phosphate acquisition and the maintenance of functional arbuscules. Genetic and physiological studies further indicate that disruption of PT4/PT11-mediated phosphate transport is closely associated with premature arbuscule degeneration and impaired symbiotic performance, highlighting a functional link between phosphate uptake, nutrient exchange, and arbuscule maintenance. In this review, we summarize current knowledge of the molecular mechanisms underlying PT4/PT11 function and regulation during AM symbiosis. We first compare mycorrhiza-specific phosphate transporters across plant species and discuss their localization, transport properties, and contributions to the mycorrhizal P_i_ uptake pathway. We then examine the multilayered regulatory network controlling PT4/PT11 expression, including the common symbiosis signaling pathway, calcium-dependent signaling, CYCLOPS–RAM1-centered transcriptional regulation, AP2/ERF-family regulators, hormone-associated regulation, and systemic phosphate-sensing pathways. Particular attention is given to the functional coupling between PT4/PT11-mediated phosphate uptake and other components of reciprocal nutrient exchange, including carbon and lipid transfer, proton extrusion, and arbuscule maintenance. We further discuss how impairment of phosphate transport through PT4/PT11 affects arbuscule integrity and the physiological conditions of the plant–fungus interface. Based on available genetic and physiological evidence, we propose a working model in which PT4/PT11-mediated phosphate uptake contributes to maintaining a favorable interfacial phosphate environment and efficient nutrient exchange, thereby supporting arbuscule function and longevity. Importantly, current evidence does not demonstrate that PT4/PT11 possess a transport-independent function or directly transmit signals through interactions with downstream signaling components. The mechanisms linking transporter activity, local phosphate status, nutrient exchange, and arbuscule maintenance therefore remain to be experimentally resolved. Finally, we highlight emerging approaches, including single-cell and spatial transcriptomics, proximity labeling, transport-defective mutants, and genome editing, that may distinguish transport-dependent from potential transport-independent functions of PT4/PT11. Understanding these mechanisms may provide a conceptual and practical foundation for exploiting AM symbiosis to improve phosphorus-use efficiency, reduce dependence on mineral phosphate fertilizers, and develop more sustainable crop production systems.

## 1 Introduction

### 1.1 Global phosphorus limitation

Phosphorus is one of the three major macronutrients essential for plant growth and development and plays a key role in energy metabolism, nucleic acid biosynthesis, membrane structure, and signal transduction. It also contributes to sugar and starch metabolism, grain formation and maturation, biological nitrogen fixation, and root system development (Zeng et al., 2026). The primary sources of phosphorus that plants obtain from the soil can be categorized into inorganic and organic phosphorus. Inorganic phosphorus is further subdivided into water-soluble, adsorbed, and mineral forms, while organic phosphorus consists mainly of inositol phosphates, phosphate esters, nucleic acids, and microbial biomass phosphorus (Huang et al., 2017).

However, the primary form of phosphorus absorbed by the vast majority of plants from the soil is inorganic phosphate; because phosphate readily forms insoluble complexes with soil metal cations, the concentration of plant-available phosphorus is often low. Low-phosphorus stress not only alters plant morphology and physiological and biochemical processes but can also affect plant growth and development, thereby limiting vegetation productivity (López-Arredondo et al., 2014). As a vital plant nutrient, phosphorus has limited reserves and is prone to soil fixation; furthermore, its runoff leads to eutrophication in water bodies. The low-use efficiency and substantial losses of phosphorus fertilizers have become major constraints on sustainable agricultural production (Li et al., 2024).

### 1.2 Arbuscular mycorrhizal fungi improve phosphate acquisition

Soil microorganisms—including bacteria, fungi, and actinomycetes—are vital components of the soil ecosystem. They decompose organic matter, transform nutrients, improve soil structure, promote plant growth, enhance plant resilience to abiotic stress, and purify the soil environment, serving as a fundamental force in maintaining natural material cycling and soil health (Chen et al., 2024a**;** Wang et al., 2024**;** Zhong et al., 2025). Over the course of long-term evolution, plants have evolved various strategies for acquiring nutrients from soil. These include direct uptake through roots and indirect acquisition through symbiotic associations with soil microorganisms (Zhao et al., 2024). Mycorrhizae are mutualistic associations between fungi and plant roots, and more than 80% of terrestrial plant species are capable of forming mycorrhizal associations. Mycorrhizal associations are broadly classified into ectomycorrhizal and endomycorrhizal types, with arbuscular mycorrhiza (AM) representing the most widespread endomycorrhizal association. The arbuscular interface within root cortical cells provides a specialized site for reciprocal nutrient exchange between the plant and fungal partners, facilitating the transport of mineral nutrients and water to the plant while simultaneously acquiring carbohydrates from the plant’s root system (Bücking and Heyser, 2001**;** Kyslynska et al., 2023**;** Harrison, 2005). Among endomycorrhizae, the most widely distributed type is known as AM symbiosis. AM symbiosis is an aseptate endomycorrhizal association characterized by the formation of specialized fungal structures, particularly arbuscules and, in some species, vesicles, within host roots (Brundrett, 2004**;** Smith and Smith, 1997). Eighty percent of vascular plants are capable of forming arbuscular mycorrhizae, which are highly branched fungal structures that develop within root cortical cells. While the arbuscular hyphae essentially fill the entire space of the cortical cell, the fungal plasma membrane does not fuse with the plant plasma membrane; instead, the plant plasma membrane forms a specialized structure, the periarbuscular membrane (PAM), that envelops the arbuscular hyphae. The interface between the arbuscular plasma membrane of the mycorrhizal fungus and the PAM of the plant is hypothesized to be the primary interface for the exchange of soluble nutrients between the two symbionts (Ivanov and Harrison, 2014**;** Cox and Tinker, 1976**) (****Figure 1**).

**Figure 1:**
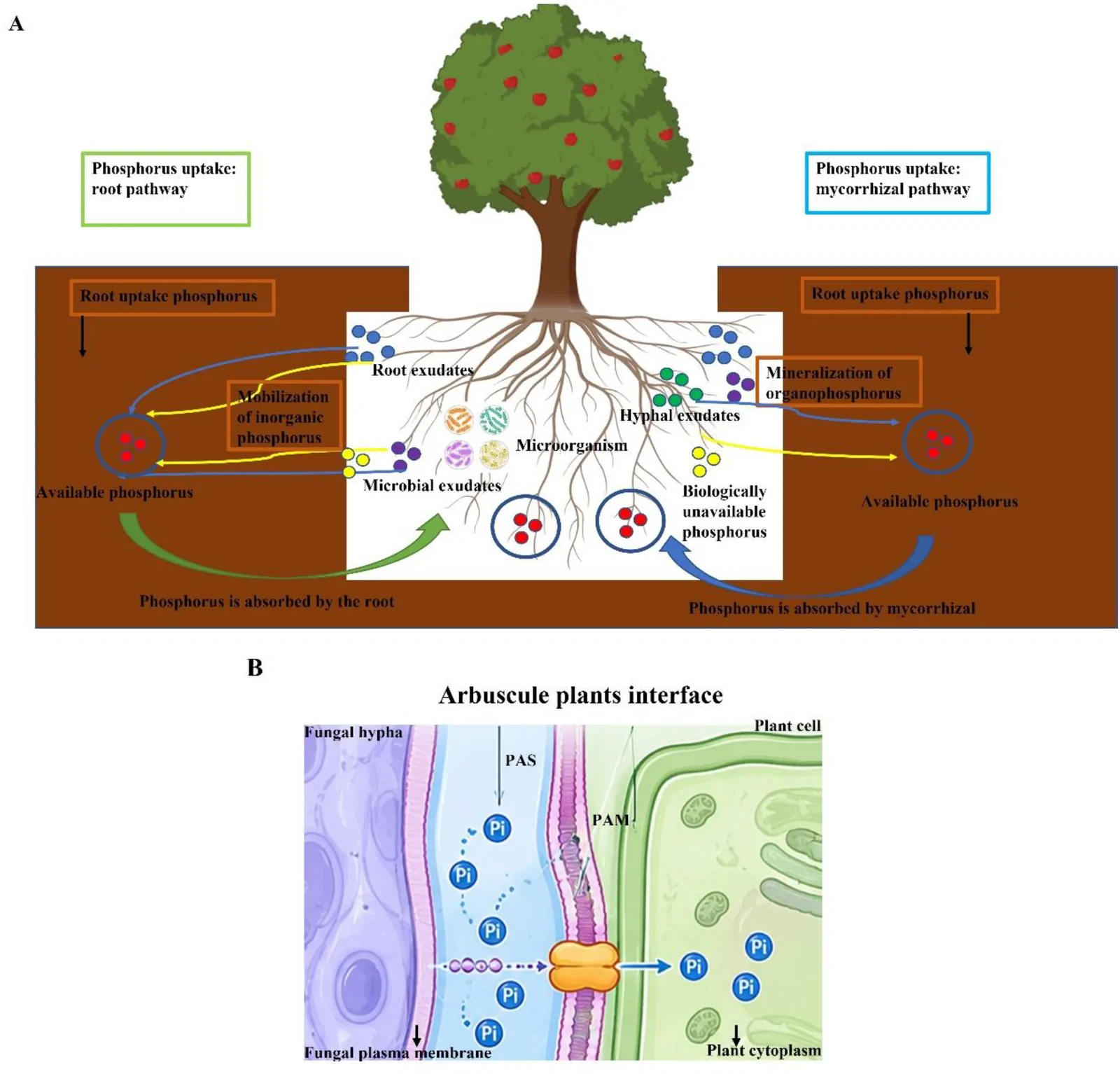
Schematic representation of plant phosphorus acquisition pathways. **(A)** Phosphorus uptake by plants occurs via two major routes: the root pathway (direct uptake by root epidermal cells and root hairs) and the mycorrhizal pathway (uptake mediated by AM fungi). In the root pathway, plants directly absorb available inorganic phosphorus from the soil solution through root epidermal cells. In the mycorrhizal pathway, extraradical hyphae of AM fungi extend beyond the rhizosphere and take up phosphorus from soil, which is then transported to intraradical hyphae and released into the periarbuscular space for subsequent plant uptake via mycorrhiza specific phosphate transporters. Additionally, soil microorganisms contribute to phosphorus mobilization through the secretion of phosphatases and organic acids, which facilitate the mineralization of organic phosphorus and the solubilization of non-bioavailable inorganic phosphorus, thereby increasing the pool of available phosphorus for both root and hyphal uptake. Root and hyphal exudates further modulate rhizosphere microbial activity and phosphorus cycling. **(B)** Arbuscule plant interface: In the mycorrhizal pathway, extra-root hyphae absorb phosphorus from the soil and transport it in the form of polyphosphates to the intra-root hyphae, where it is hydrolyzed at the branch points to release inorganic phosphorus (P_i_) into the periarbuscular space (PAS). Mycorrhiza-induced phosphate transporters located on the periarbuscular membrane (PAM) of plant root epidermal cells, transport P_i_ from the PAS into the plant cytoplasm. Image materials are generated from https://www.biogdp.com.

AM fungi form symbiotic relationships with more than two-thirds of plant species in terrestrial ecosystems. The extraradical hyphae of AM fungi extend beyond the rhizosphere, expanding the plant’s nutrient-acquisition zone well past the root surface; their most critical function is facilitating the uptake of water and mineral nutrients, particularly inorganic phosphate, which is essential for plant growth and development. Although AM fungi possess some phosphatase genes, their ability to mineralize organic phosphorus is limited compared to ectomycorrhizal fungi, their extraradical hyphae can modify the rhizosphere and hyphosphere environments and interact with associated microbial communities, potentially contributing to phosphorus mobilization (Li et al., 1991**;** Zhang et al., 2022**;** Jiang et al., 2021). AM fungi utilize high-affinity phosphate transporters—encoded by the fungi themselves—to take up soluble phosphorus from the soil, transport it into the fungal cytoplasm, and store it in vacuoles. These fungal phosphate transporters belong to the major facilitator superfamily (MFS). Three phosphate transporters, GvPT, GiPT, and GmosPT, have been cloned from *Glomus versiforme*, *G. intraradices*, and *G. mosseae*, respectively; among them, *GmosPT* is highly expressed in intraradical hyphae (Harrison and Van Buuren, 1995**;** Maldonado-Mendoza et al., 2001**;** Benedetto et al., 2005**;** Ansari et al., 2026). AM fungi acquire inorganic phosphate from the soil through extraradical hyphae and transport it toward intraradical structures, where Pi is polymerized into polyphosphate (polyP) and translocated to arbuscules. Within arbuscules, polyP is hydrolyzed to release inorganic phosphate, which must subsequently be exported across the fungal membrane into the periarbuscular space (PAS) before being taken up by plant PHT1 phosphate transporters such as PT4 and PT11. Although the complete molecular mechanism of fungal phosphate unloading remains unresolved, recent studies have identified several candidate transport systems involved in this process. In particular, RiPT7, an SPX (SYG1/Pho81/XPR1) domain-containing phosphate transporter from *Rhizophagus irregularis*, is expressed in arbuscules and intraradical mycelia and can mediate phosphate transport across the fungal plasma membrane. Silencing of RiPT7 impairs arbuscule development and symbiotic phosphate delivery, supporting a role for this transporter in fungal phosphate homeostasis and phosphate transfer at the symbiotic interface (Xie et al., 2022). In addition, fungal PHO1/SYG1-type proteins, including RiPHO1, RcSYG1 from *Rhizophagus clarus*, and GmPHO1 from *Gigaspora margarita*, have been proposed to contribute to phosphate export from arbuscules through an exocytotic pathway (Ezawa and Saito, 2018**;** Duan et al., 2024). Thus, current evidence supports a model in which fungal phosphate unloading may involve multiple, potentially condition-dependent pathways, including SPX-domain-containing transporters and PHO1/SYG1-type proteins, rather than a single dedicated exporter. Following release into the PAS, phosphate is taken up across the periarbuscular membrane (PAM) by plant PHT1 transporters such as PT4 and PT11. Thus, PT4/PT11-mediated phosphate uptake represents the plant-side step of a broader fungal-to-plant phosphorus transfer pathway (Karandashov and Bucher, 2005).

Consequently, plants can take up phosphorus from the soil directly through the root epidermis and root hairs, as well as via the mycorrhizal pathway (Qi et al., 2022**;** Shi et al., 2021**;** Smith et al., 2011). Under low-phosphorus stress, plants expand their root absorptive surface area to acquire more phosphorus by increasing the number and length of root hairs and developing finer, longer roots (Lynch, 2011). Experimental data on the arbuscular mycorrhizal symbiosis indicate that mycorrhizae significantly promote phosphorus uptake by the plant (Qi et al., 2022**;** Shi et al., 2021**;** Smith et al., 2011). This is because hyphae can reach soil areas inaccessible to plant roots, helping the plant expand its phosphorus absorption area. Therefore, under phosphorus-deficient conditions, plants are more inclined to establish mycorrhizal symbioses with mycorrhizal fungi to enhance their ability to acquire phosphorus from the soil; conversely, high phosphorus levels inhibit the development of arbuscular mycorrhizae (Balzergue et al., 2010**;** Paries and Gutjahr, 2023). Furthermore, the mechanisms by which ectomycorrhizal and AM fungi promote plant phosphorus uptake differ. AM fungi possess genes encoding multiple phosphatases that can promote the activation of organophosphates, but their contribution to organophosphate mineralization is generally considered more limited than that of many ectomycorrhizal fungi. (Joner et al., 2000**;** Rosling et al., 2016). Therefore, ectomycorrhizal fungi primarily hydrolyze organic phosphorus by secreting phosphatases, whereas AM fungi generally exhibit lower phosphatase activity and rely more on associated phosphate-solubilizing microorganisms.

### 1.3 PT4/PT11 phosphate transporter in an arbuscular mycorrhizal symbiosis system

During AM fungal-mediated phosphate transport, gene regulation plays a pivotal role in both the AM fungi and the plant host, thereby facilitating the efficient transfer of soil phosphate into the plant. Similar to the cases of endomycorrhizae and ectomycorrhizae, high-affinity phosphate transporters encoded by genes such as *MtPT4* in *Medicago truncatula*, *OsPT11* in *Oryza sativa*, *LjPT4* in *Lotus japonicus*, *ZmPT6* in *Zea mays*, and *SlPT4* in *Solanum lycopersicum* mediate phosphate transport across the symbiotic interface. These transporters belong to the phosphate transporter 1 (PHT1) family (Chen et al., 2024b**;** Rui et al., 2022**;** Javot et al., 2007a**;** Javot et al., 2007b**;** Walder et al., 2015**;** Volpe et al., 2016). The expression of these phosphate transporter genes is strongly induced during AM symbiosis, the transporters localize to the PAM of plant root cortical cells containing arbuscules and actively take up phosphate released by AM fungi through proton-coupled phosphate transport (Xie et al., 2013**;** Willmann et al., 2013). Functionally, these transporters constitute major components of the plant-side mycorrhizal phosphate uptake pathway. After AM fungi transport phosphate predominantly in the form of polyphosphate through their hyphae to the arbuscules, polyphosphate is hydrolyzed to release inorganic phosphate. The subsequent unloading of phosphate from the fungal arbuscule into the PAS is thought to involve multiple fungal transport systems (Xie et al., 2016**;** Kobae, 2019). Recent evidence implicates the SPX-domain-containing transporter RiPT7 in this process, while PHO1/SYG1-type proteins such as RiPHO1 and RcSYG1 have also been proposed to contribute to fungal phosphate export (Ezawa and Saito, 2018**;** Xie et al., 2022**;** Duan et al., 2024). Once phosphate is released into the PAS, plant PHT1 transporters such as MtPT4 and OsPT11, localized on the PAM, mediate its uptake into the arbuscule-containing cortical cells. Thus, PT4/PT11 represent the plant-side uptake component of a broader fungal-to-plant phosphate transfer pathway that includes fungal phosphate mobilization, unloading into the PAS, and subsequent plant uptake.

This process is crucial for maintaining the stability of the plant-arbuscular mycorrhizal symbiosis; studies have shown that knocking out or suppressing the genes encoding *MtPT4* or *OsPT11* not only blocks the uptake of fungus-derived phosphate but also leads to the premature degradation of arbuscular structures and a sharp decline in symbiotic efficiency, indicating that the function of these phosphate transporters is indispensable for maintaining the symbiotic relationship (Choi et al., 2018**;** Jia et al., 2025). The expression of these transporters is coordinately regulated by plant phosphate-starvation signaling pathways and symbiotic signaling networks. Their gene promoter regions typically contain PHR transcription factor binding sites (P1BS) characterized by the core sequence GNATATNC as well as mycorrhiza-specific transcription factor binding sequences (MYCS) and AW-box binding sequences (AAAGATAT…) and so on. This ensures their precise, induced expression under conditions of low phosphate availability and fungal colonization (Ansari et al., 2026**;** Chen et al., 2011). It is noteworthy that although this type of mycorrhiza-inducible PHT1 transporter has been identified in both monocots and dicots, the phylogenetic relationships of their homologous proteins are not entirely conserved across different species, suggesting that different plants may have evolved distinct molecular strategies to adapt to symbiotic phosphate uptake (Paszkowski et al., 2002**;** Karandashov et al., 2004). The expression of mycorrhizal phosphate transporter genes is linked to phosphate levels, with high phosphate concentrations inhibiting their expression (Nagy et al., 2009). Xu et al. used *Slpt4* mutants in tomato to further demonstrate that *SlPT4* is specifically induced by mycorrhizal colonization, the loss of *SlPT4* function cannot be compensated for by other phosphate transporter genes (Xu et al., 2007). Pumplin et al. and Kobae et al. utilized existing organelle-specific fluorescent marker proteins to further analyze cellular states during arbuscule maintenance and degeneration. They observed that MtPT4-GFP and OsPT11-GFP localized to the fine branches of mature arbuscules but were undetectable in young or degenerating arbuscules; this established that these mycorrhiza-specific phosphate transporters (MtPT4 and OsPT11) undergo degradation alongside the arbuscules, suggesting a potential link between mycorrhizal phosphate transporters and the processes of arbuscule maintenance and degeneration (Pumplin and Harrison, 2009**;** Kobae and Hata, 2010).

The expression of these phosphate transporter genes is regulated by the SPX-PHR signaling pathway within the plant; this pathway modulates the expression of phosphate starvation response genes by sensing intracellular phosphate status. SPX proteins sense intracellular soluble inositol polyphosphates (InsPs) and regulate the activity of PHR transcription factors to induce the expression of *phosphate starvation-induced* (*PSI*) genes, thereby enhancing the plant’s adaptability to low phosphate conditions (Jung et al., 2018**;** Yang et al., 2024**;** Zhou et al., 2021). Under low phosphate conditions, the binding affinity of SPX for PHR2 is significantly reduced, leading to a rapid increase in PHR2 protein levels; this facilitates the binding of PHR2 to *P1BS* elements within the promoters of phosphate transporter genes and activates the expression of genes related to phosphate transport, thereby enhancing the plant’s capacity for phosphate uptake and utilization (Wang et al., 2014). However, under high-phosphate conditions, SPX proteins bind to PHRs, sequestering them or inhibiting their DNA-binding activity, thereby suppressing the expression of *PSI* genes (Puga et al., 2014**;** Wang et al., 2014**;** Ge et al., 2026**;** Mangalakkadan et al., 2026).

In summary, PT4/PT11 are key executors of phosphate uptake at the plant–fungus symbiotic interface. Their activity is essential for efficient mycorrhizal phosphate acquisition and is closely associated with the maintenance of functional arbuscules. Loss of these transporters impairs phosphate uptake and is accompanied by premature arbuscule degeneration, demonstrating a strong functional connection between phosphate transport and symbiotic maintenance. Rather, current findings support the view that PT4/PT11-mediated phosphate transport may influence arbuscule function indirectly by shaping the nutritional and physiological conditions of the symbiotic interface. Defining the molecular mechanisms that connect transporter activity to arbuscule maintenance therefore remains an important challenge for future research.

In this review, we distinguish between “mycorrhiza-specific” and “mycorrhiza-inducible” phosphate transporters. The former refers to transporters whose expression is largely restricted to mycorrhizal tissues or arbuscule-containing cells, whereas the latter refers to transporters whose expression is strongly induced by AM colonization but may also occur at detectable levels in non-mycorrhizal roots. Because these categories can overlap depending on species, tissue, developmental stage, and experimental conditions, we use the terms according to the available expression evidence for each transporter.

### 1.4 Symbiotic signal transduction pathway of arbuscular mycorrhizal fungi

AM fungal symbiosis is a form of endosymbiosis. The initiation of AM symbiotic signaling begins with the secretion of molecules such as strigolactones (SLs) and flavonoids from plant roots into the rhizosphere under conditions of nutrient deficiency (Akiyama et al., 2005). SLs not only act as chemical attractants to stimulate the germination and hyphal branching of AM fungal spores but also create conditions for the establishment of symbiosis by altering the rhizosphere microenvironment (Rodriguez-Garcia and Müller, 2026**;** Samanta et al., 2025). In response, AM fungi release signaling molecules known as Myc factors, the primary components of which are short-chain chitin oligomers (COs) and lipochitin-oligosaccharides (LCOs) (Paszkowski et al., 2006). Plant root epidermal cells perceive these fungal signals through members of the LysM-RLK (Lysin motif-containing receptor-like kinase) family. Following perception of fungal Myc factors by LysM receptor-like kinases, receptor-associated signaling involving SYMRK/DMI2 activates downstream components, including the nuclear ion channels DMI1/CASTOR/POLLUX, leading to characteristic nuclear Ca²^+^ oscillations. These Ca²^+^ signatures are decoded by the calcium/calmodulin-dependent protein kinase CCaMK/DMI3, which, together with CYCLOPS/IPD3 and DELLA proteins, promotes transcriptional activation of RAM1 and other symbiosis-associated genes. Specifically, CCaMK phosphorylates the downstream regulator CYCLOPS, which activates a transcriptional regulatory network involving the GRAS-family transcription factor RAM1 (Required for Arbuscular Mycorrhization 1), thereby initiating the expression of a series of Myc-induced and symbiosis-related genes within the nucleus, which in turn drives fungal infection and the formation of arbuscular structures. This pathway is commonly referred to as the arbuscular mycorrhizal symbiosis signaling pathway (Stracke et al., 2002**;** Díaz et al., 2025**;** Genre and Russo, 2016). Some studies indicate that the proteins CCaMK, CYCLOPS, and DELLA cooperatively form a functional protein complex within the symbiotic signaling pathway. This complex directly binds to and activates the promoter region of the key downstream transcription factor RAM1, thereby initiating the gene expression program essential for arbuscular mycorrhizal symbiosis. Specifically, CCaMK is activated upon sensing nuclear calcium oscillations triggered by symbiotic signals and subsequently phosphorylates CYCLOPS. Phosphorylated CYCLOPS physically interacts with DELLA proteins (repressors of gibberellin signaling), and the three components assemble into a transcriptional regulatory complex that specifically recognizes cis-acting elements of *RAM1*. This mechanism reveals how the crosstalk among calcium signaling, phosphorylation modification, and hormonal signaling converges at a core transcriptional node, ensuring the efficient initiation of mycorrhizal symbiosis by the plant under favorable conditions (Pimprikar et al., 2016**;** Pimprikar and Gutjahr, 2018).

Recent research suggests that signal transduction is not limited to the initial stages of symbiosis establishment; continuous signal exchange is also required during the development and functional maintenance of arbuscules within cortical cells. The PAS is the interface between plants and fungi; it serves not only as a site for nutrient exchange but also as a hub for continuous signal communication mediated by signal peptides, cell wall components, and extracellular vesicles. Furthermore, the concept of a common symbiotic signaling pathway is being re-evaluated: while core components are shared between AM and root nodule symbioses, growing evidence suggests that pathway outputs are symbiont-specific and that bypass or parallel signaling pathways may exist. Concurrently, plants have evolved sophisticated negative regulatory mechanisms to restrict excessive symbiotic development, thereby ensuring a balance between nutrient investment and the benefits gained (Rodriguez-Garcia and Müller, 2026**;** Genre and Russo, 2016**;** Gutjahr et al., 2008).

While previous reviews have largely focused on phosphate transporters or AM nutrition, this article highlights the pivotal roles of PT4 and PT11 as a functional association in coordinating nutrient exchange, arbuscule maintenance, feedback signaling, and the maintenance of symbiosis. In summary, signal transduction in AM symbiosis constitutes a complex, multi-stage, and multi-component network; it begins with chemical dialogue in the rhizosphere and proceeds through perception by LysM receptors and nuclear signal transduction via the Common Symbiosis Signaling Pathway, ultimately leading to cellular reprogramming and the establishment of symbiotic structures. A deeper understanding of this pathway not only unveils the evolutionary mysteries of plant-fungal interactions but also provides molecular targets and a theoretical basis for leveraging AM fungi to enhance crop nutrient efficiency and stress tolerance (**Figure 2**).

**Figure 2:**
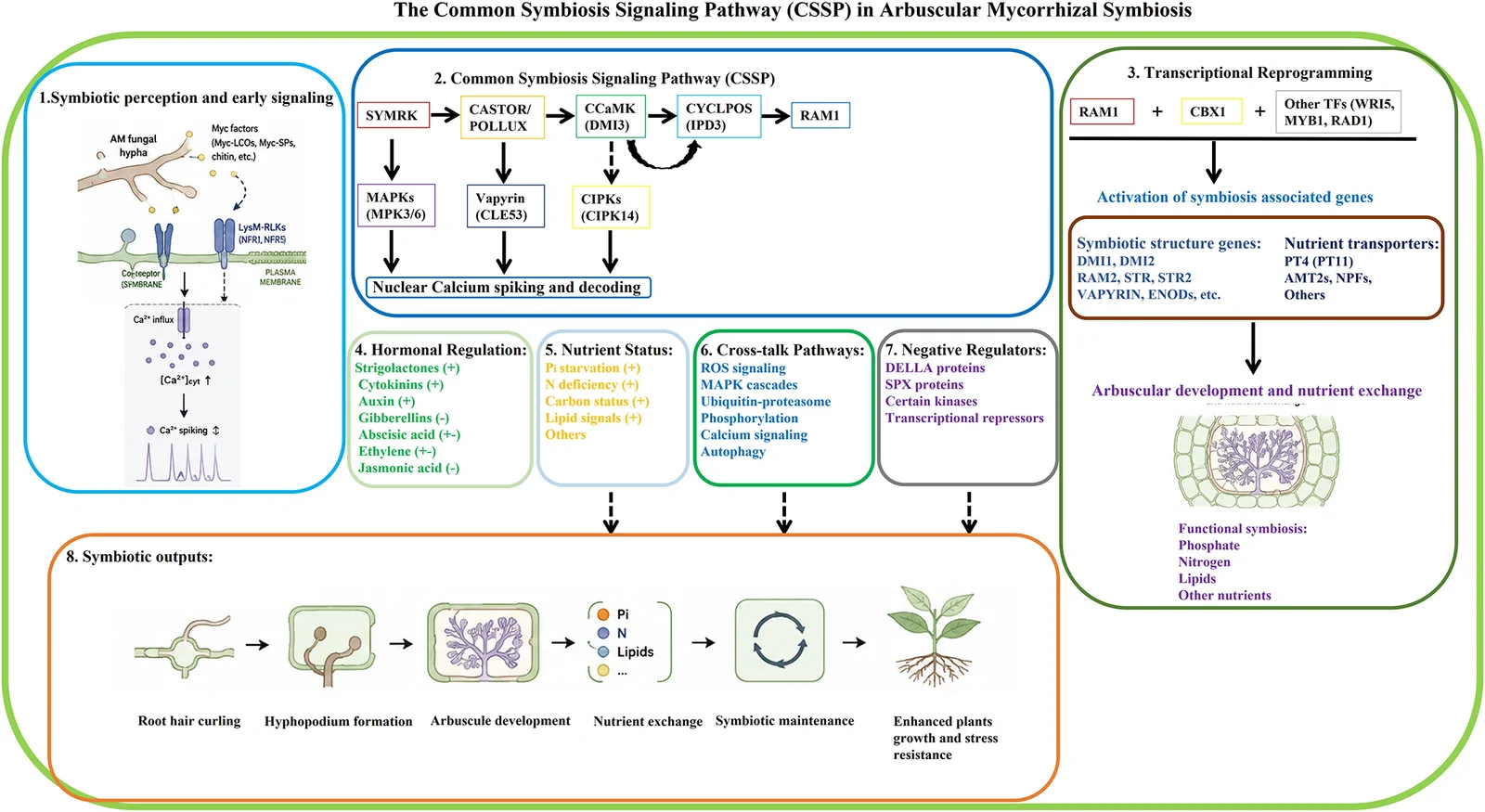
Common symbiosis signaling pathway underlying arbuscular mycorrhizal (AM) symbiosis. AM fungal signals, including mycorrhizal lipochitooligosaccharides (Myc-LCOs) and short-chain chitin oligomers (Myc-COs), are perceived by host LysM receptor-like kinases (RLKs) at the plasma membrane, initiating the common symbiosis signaling pathway (CSSP). Signal perception activates downstream components, including SYMRK/DMI2, MAPKs, and nuclear-localized signaling proteins such as CASTOR and POLLUX, resulting in characteristic nuclear Ca²^+^ oscillations. The Ca²^+^ signal is decoded by the calcium/calmodulin-dependent protein kinase CCaMK/DMI3, which activates the transcriptional regulator CYCLOPS/IPD3 and downstream symbiotic transcriptional programs. These signaling events converge on key transcription factors, including RAM1, CBX1, WRI5, and other AM-associated regulators, to activate genes required for arbuscule formation, nutrient transport, and symbiotic nutrient exchange. The CSSP is further integrated with hormonal signals, nutrient status, ROS/MAPK signaling, phosphorylation, ubiquitin–proteasome pathways, and negative regulatory mechanisms involving DELLA proteins, SPX proteins, and transcriptional repressors. Coordinated transcriptional reprogramming ultimately promotes arbuscule development, phosphate and nitrogen acquisition, lipid transfer, and establishment of a functional and sustainable AM symbiosis. PT4/PT11 are important AM-specific phosphate transporters within this downstream symbiotic transcriptional network and mediate phosphate uptake across the periarbuscular membrane. Abbreviations: AM, arbuscular mycorrhiza; CCaMK, calcium/calmodulin-dependent protein kinase; CSSP, common symbiosis signaling pathway; LCOs, lipochitooligosaccharides; RLK, receptor-like kinase; TFs, transcription factors; P_i_, inorganic phosphate; N, nitrogen.

## 2 Evolution and conservation of AM-specific phosphate transporters

### 2.1 Phosphate transporter 1 family

Through fungal hyphal networks, AM symbiosis facilitates the uptake of soil phosphorus and its transport to the arbuscular interface within root cortical cells; at this interface, members of the PHT1 phosphate transporter family mediate phosphate uptake across the PAM into the host cortical cell (Javot et al., 2007a**;** Javot et al., 2007b**;** Ahmed et al., 2025).

Plant PHT1 proteins are members of the major facilitator superfamily (MFS) of membrane transport proteins and function as proton-coupled phosphate transporters. PHT1 proteins mediate high-affinity phosphate (P_i_) uptake in plant cells. These proteins exhibit a characteristic structure of 12 transmembrane helices; their function relies on the proton motive force, utilizing the proton gradient across the plasma membrane to cotransport P_i_ ions into the cytoplasm (Bayle et al., 2011**;** Zhou et al., 2025**;** Li et al., 2019). Nine PHT1 family members have been identified in the model plant *Arabidopsis thaliana*, and thirteen in *Oryza sativa*. Based on sequence similarity, expression patterns, and functional differences, PHT1 members can be further categorized into several subgroups. Notably, members closely associated with AM symbiosis primarily belong to specific subclades; their transcription is specifically induced by AM fungal colonization, and their spatiotemporal expression dynamics are highly synchronized with the progression of arbuscule maintenance. Their transcription is strongly induced during arbuscule maintenance and typically reaches its highest levels during arbuscule maturation, followed by a decline as arbuscules enter senescence. This dynamic expression pattern is closely associated with their role in phosphate exchange at the symbiotic interface (Sun et al., 2017**;** Nagarajan et al., 2011**;** McGaley et al., 2026).

### 2.2 AM symbiosis-induced PHT1 members and functional validation

In *Medicago truncatula*, MtPT4 was the first gene definitively identified as an AM symbiosis-specific phosphate transporter. It is expressed exclusively in cortical cells containing arbuscules, and the encoded protein localizes to the PAM, the plant plasma membrane surrounding the arbuscular branches, thereby positioning the transporter at the key site of nutrient exchange. In loss-of-function *MtPT4* mutants, although AM fungi can still successfully colonize roots and form intact arbuscules, these arbuscules undergo premature degeneration, and the transfer and subsequent uptake of fungus-derived phosphate at the symbiotic interface are severely impaired, resulting in a severe reduction or loss of the mycorrhizal contribution to plant phosphorus nutrition under the tested conditions. This pivotal experimental evidence established the irreplaceable role of this PHT1 family member as an interfacial phosphate unloader (Harrison et al., 2002**;** Javot et al., 2011). In *Lotus japonicus*, *LjPT4* encodes a phosphate transporter involved in AM symbiosis; its primary functions involve phosphate nutrient uptake during AM symbiosis and the regulation of root system development. During arbuscular mycorrhizal symbiosis, it localizes to the membrane of cells containing arbuscules and is responsible for acquiring inorganic phosphate released by arbuscular mycorrhizal fungi, thereby providing nutrients to the host plant. This gene plays a key role in maintaining the normal morphology and developmental structure of fungal arbuscules within the root (Volpe et al., 2016**;** Rui et al., 2022**).**

OsPT11 is a phosphate transporter gene in *Oryza sativa* that primarily mediates phosphate uptake and transport and plays a crucial role in AM symbiosis. The protein localizes to the plasma membrane of cortical cells enveloping the arbuscules; furthermore, *OsPT11* activation is regulated specifically by mycorrhizal symbiotic signals. OsPT11 is a major contributor to the mycorrhizal phosphate uptake pathway in rice, facilitating nutrient acquisition in low-phosphorus soils (Paszkowski et al., 2002**;** Mitra et al., 2026). Similar AM- inducible PHT1 members, including *SlPT4* and *SlPT5* in tomato, have subsequently been identified in several plant species. In tomato, these transporters are strongly induced following AM fungal colonization and contribute to phosphate acquisition during symbiosis. Furthermore, as key molecular markers of tomato arbuscular mycorrhizal symbiosis, the expression levels of these two genes reflect the activation and regulatory status of symbiotic signaling between the plant and the beneficial fungi (Rui et al., 2023**;** Chen et al., 2024b). Similar AM-specific PHT1 members, including *StPT3* and *StPT4* in potato, have also been identified. These genes encode high-affinity phosphate transporters that are induced following AM fungal colonization and arbuscule formation, thereby facilitating phosphate acquisition at the symbiotic interface (Loth-Pereda et al., 2011**;** Nagy et al., 2005). In summary, the functional module comprising AM-specific and AM-inducible PHT1 members is highly conserved in angiosperms (**Table 1**).

**Table 1:** Mycorrhiza-specific and mycorrhiza-inducible phosphate transporters in plant endomycorrhizae.

Gene name	Species	PHT1 subgroup	Serial Number	Gene expression pattern	Reference
*MtPT4*	*Medicago truncatula*	PHT1	AAM76744	Mycorrhiza-specific	(Harrison et al., 2002; Javot et al., 2007a; Pumplin and Harrison, 2009)
*LjPT4*	*Lotus japonicus*	PHT1	BAE93353	Mycorrhiza-inducible	(Maeda et al., 2006; Guether et al., 2009 )
*OsPT11*	*Oryza sativa*	PHT1	AAN39052	Mycorrhiza-specific	(Paszkowski et al., 2002; Kobae and Hata, 2010; Güimil et al., 2005)
*OsPT13*	*Oryza sativa*	PHT1	AAN39054	Mycorrhiza-inducible	(Paszkowski et al., 2002; Güimil et al., 2005)
*NtPT3*	*Nicotiana tabacum*	PHT1	EF091669	Mycorrhiza-inducible	(Chen et al., 2007)
*NtPT4*	*Nicotiana tabacum*	PHT1	EF091672	Mycorrhiza-inducible	(Chen et al., 2007)
*NtPT5*	*Nicotiana tabacum*	PHT1	EF091675	Mycorrhiza-inducible	(Chen et al., 2007)
*Pht1; 6*	*Zea mays*	PHT1	CAH25731	Mycorrhiza-inducible	(Glassop et al., 2005; Nagy et al., 2006)
*Pht1; 8*	*Triticum aestivum*	PHT1	CAH25730	Mycorrhiza-specific	(Glassop et al., 2005)
*Pht1; myc*	*Hordeum vulgare*	PHT1	AAO72440	Mycorrhiza-inducible	(Rae et al., 2003; Glassop et al., 2005)
*CfPT3*	*Capsicum frutescens*	PHT1	EF091667	Mycorrhiza-inducible	(Chen et al., 2007)
*CfPT4*	*Capsicum frutescens*	PHT1	EF091670	Mycorrhiza-inducible	(Chen et al., 2007)
*CfPT5*	*Capsicum frutescens*	PHT1	EF091673	Mycorrhiza-inducible	(Chen et al., 2007)
*SmPT3*	*Solanum melongena*	PHT1	EF091668	Mycorrhiza-inducible	(Chen et al., 2007)
*SmPT4*	*Solanum melongena*	PHT1	EF091671	Mycorrhiza-inducible	(Chen et al., 2007)
*SmPT5*	*Solanum melongena*	PHT1	EF091674	Mycorrhiza-inducible	(Chen et al., 2007)
*StPT3*	*Solanum tuberosum*	PHT1	CAC87043	Mycorrhiza-inducible	(Nagy et al., 2005; Rausch et al., 2001)
*StPT4*	*Solanum tuberosum*	PHT1	AAW51149	Mycorrhiza-specific	(Nagy et al., 2005)
*StPT5*	*Solanum tuberosum*	PHT1	AAX85195	Mycorrhiza-specific	(Nagy et al., 2005)
*LePT3*	*Lycopersicon esculentum*	PHT1	AY804011	Mycorrhiza-inducible	(Nagy et al., 2005)
*LePT4*	*Lycopersicon esculentum*	PHT1	AAX85192	Mycorrhiza-specific	(Nagy et al., 2005)
*LePT5*	*Lycopersicon esculentum*	PHT1	AAX85194	Mycorrhiza-inducible	(Nagy et al., 2005)
*SlPT3*	*Solanum lycopersicum*	PHT1	AY486010	Mycorrhiza-inducible	(Rui et al., 2023)
*SlPT4*	*Solanum lycopersicum*	PHT1	AY486011	Mycorrhiza-inducible	(Rui et al., 2023)
*SlPT5*	*Solanum lycopersicum*	PHT1	AY486012	Mycorrhiza-inducible	(Rui et al., 2023)

### 2.3 Coupling of PHT1-mediated phosphate transport and carbon-phosphate exchange

Phosphate transfer during AM symbiosis is not a unidirectional supply of nutrients; rather, it is a tightly coordinated and mutually beneficial process. The plant relies on the fungus to acquire additional nutrients, while in return, it provides the fungus with carbon sources to sustain its vital activities (Chiu and Paszkowski, 2019**;** Javot et al., 2007a**;** Javot et al., 2007b). Phosphorus absorbed from the soil by the fungus is primarily transported to the arbuscules in the form of polyphosphate via cytoplasmic streaming within the hyphae; there, it is depolymerized into releasable inorganic phosphate. On the plant side, PHT1 proteins are responsible for the efficient uptake of phosphate from this interface space into cortical cells. Notably, this phosphate uptake process is coupled via signaling mechanisms to the transfer of photosynthetic products to the fungus: when the plant’s phosphate status is sufficient, the host actively downregulates the expression of AM-specific PHT1 and reduces carbon allocation, thereby establishing a negative feedback loop that ensures the economic sustainability of the symbiosis (Solaiman et al., 1999**;** Karandashov and Bucher, 2005). In recent years, the transcription factor PHR2 and GRAS family proteins RAD1 have been shown to directly regulate PHT1 gene transcription, integrating the plant’s overall phosphorus nutritional status with symbiotic phosphate transport activity within a unified regulatory network (Rey et al., 2017**;** Shi et al., 2021).

### 2.4 Coordinated nutrient transport across the periarbuscular interface

The PAM is not a dedicated phosphate transport boundary but a specialized plant-derived membrane that accommodates multiple transport processes required for reciprocal nutrient exchange during AM symbiosis. PT4/PT11-mediated phosphate uptake therefore operates within a broader transport network that coordinates the movement of phosphorus, nitrogen, carbon, and other metabolites between the plant and fungal partners. Although the molecular components of this network are not yet fully defined, current evidence indicates that several classes of transport proteins are differentially localized or induced in arbuscule-containing cells, consistent with specialized nutrient exchange across the arbuscular interface (Chiu and Paszkowski, 2019**;** Javot et al., 2007b).

On the plant side, AM-associated phosphate transporters such as PT4 and PT11 mediate the uptake of inorganic phosphate released by the fungal partner into the peri-arbuscular space. Nitrogen exchange is also an important component of AM symbiosis. Ammonium transporters, including AM-associated AMT family members such as AMT3;1 in some plant species, have been implicated in the uptake of ammonium supplied by AM fungi (Rui et al., 2022**;** Chen et al., 2018). In parallel, sugar transporters of the SWEET family contribute to the transfer of plant-derived carbohydrates toward colonized cortical cells and the fungal interface. Lipid transfer represents another major component of the reciprocal exchange system, with plant ABCG-type lipid transporters, including STR/STR2, contributing to the delivery of fatty acids and related lipid compounds required by AM fungi (Kryukov et al., 2021**;** Wipf et al., 2019). These transport systems are not necessarily co-regulated by a single pathway, but their coordinated activity provides a molecular basis for the bidirectional exchange of nutrients across the arbuscular interface.

The fungal partner also possesses specialized transport and metabolic systems that contribute to nutrient acquisition and delivery. Extraradical hyphae acquire phosphate from the soil through fungal phosphate transporters and translocate phosphorus toward intraradical structures. Within arbuscules, fungal phosphate is associated with polyphosphate metabolism and can subsequently be converted into inorganic phosphate that becomes available for transfer to the host. The precise mechanisms and subcellular sites of phosphate release remain incompletely resolved, and fungal phosphate mobilization is likely to involve coordinated processes of polyphosphate turnover, phosphate transport, and membrane-associated metabolism (Chiu and Paszkowski, 2019**;** Javot et al., 2007b**;** MacLean et al., 2017). Thus, plant PT4/PT11 activity represents the plant-side uptake step of a larger trans-symbiotic phosphate transfer pathway rather than an isolated transport event.

Importantly, the different transport processes at the PAM should be considered functionally coupled rather than necessarily forming a single physical transporter complex. Phosphate uptake, nitrogen acquisition, carbon transfer, and lipid delivery are metabolically interconnected and collectively influence the nutritional state of both symbiotic partners. Perturbation of one component may therefore have consequences for other exchange processes, although the extent and direction of such cross-regulation remain context-dependent. Within this broader framework, PT4/PT11 can be viewed as key components of the plant-side phosphate uptake machinery whose activity is functionally coupled to other nutrient exchange processes at the arbuscular interface.

### 2.5 Synergistic action of multiple PHT1 members in mycorrhizal symbiosis

In most plant genomes, multiple PHT1 members are induced by AM symbiosis, exhibiting a combination of functional redundancy and specialized division of labor. In tomato, the double knockout of *Slpt4* and *Slpt5* more completely abolishes the benefits of AM-mediated phosphate uptake, revealing an additive functional effect among family members. This mechanism of functional redundancy ensures the continuity of symbiotic phosphate transport under stress or mutation and implies that achieving desired crop improvements may require simultaneously targeting multiple PHT1 members in breeding programs (Rui et al., 2023**;** Chen et al., 2024b). In *Oryza sativa*, *OsPT11* and *OsPT13* are expressed in arbuscule-containing cells, *Ospt11* and *Ospt13* mutants display significant defects in both phosphate uptake and arbuscule maintenance (Paszkowski et al., 2002**;** Yang et al., 2012**;** Mitra et al., 2026).

In summary, as key executors of phosphate transport at the plant-fungus symbiotic interface, AM-specific PHT1 members constitute important components of the plant-side phosphate uptake pathway during AM symbiosis. Research on PHT1 has evolved from the functional characterization of single genes—starting with the discovery of MtPT4 in *Medicago truncatula* to in-depth investigations into phosphate transport regulatory networks, carbon-phosphate coupling mechanisms, and patterns of functional redundancy. Future research will focus on the mechanisms regulating PHT1 protein activity and the targeted utilization of these proteins in breeding crops with high phosphate-use efficiency, thereby providing molecular solutions to reduce chemical phosphate fertilizer inputs and achieve sustainable agriculture.

### 2.6 AM-specific promoter regulation: cis-elements and trans-acting factors

During the establishment of AM symbiosis, plant root cortical cells undergo profound transcriptional reprogramming, involving the specific activation or upregulation of hundreds of symbiosis-related genes. The spatiotemporal expression of these genes relies on the precise recognition and synergistic interaction between cis-acting elements within their promoter regions and corresponding transcription factors. Deciphering the regulatory codes of AM-specific promoters is an important component of understanding how plants precisely control symbiotic developmental programs and provides the molecular foundation for optimizing symbiotic benefits through genetic engineering.

Studies using *Medicago truncatula* as a model system indicate that AM-induced genes can be categorized into two subclasses based on their spatial expression patterns: one subclass is expressed specifically in cortical cells containing arbuscules (the phosphate transporter gene *MtPT4* and the serine carboxypeptidase gene *MtScp1*), while the other exhibits expression in arbuscule-containing cells as well as weaker expression in adjacent cell layers (glutathione S-transferase *MtGst1* and β-1,4-endoglucanase *MtCel1*). These differences in expression patterns suggest the existence of at least two signal activation mechanisms: one that activates arbuscule-specific genes in a cell-autonomous manner, and another that regulates the expression of the second subclass of genes in a local, non-cell-autonomous manner. Promoter-reporter gene fusion experiments reveal that relatively small upstream regions (typically 150–300 bp) are sufficient to drive AM-specific transcription, implying that cis-regulatory elements are highly concentrated within the proximal promoter region (Harrison et al., 2002**;** Küster et al., 2007).

Transcription factors from numerous families regulate target gene expression by recognizing specific DNA sequence motifs within promoters (Huang et al., 2021**;** Ma et al., 2022**;** Ma and Hu, 2024**;** Ma et al., 2024**;** Ma et al., 2025). Research on AM promoters has identified multiple functional cis-elements. The P1BS is one of the most extensively studied elements. It serves as the recognition target for the PHR transcription factor family of phosphate-responsive transcription factors. At least one copy of the P1BS element has been identified in the promoters of AM-specific phosphate transporter genes across rice, tomato, and various legumes. Functional analyses have demonstrated that P1BS is essential for the expression of AM-related genes; mutation of this element completely abolishes the expression of reporter genes in mycorrhiza colonized roots (Sega and Pacak, 2019**;** Ruan et al., 2015). PHRs activate the expression of key AM genes by binding to P1BS under low phosphate conditions, such as *PT11*, *CCD7*, and *CERK1*. Conversely, SPX proteins inhibit the nuclear localization and DNA-binding activity of PHRs, thereby suppressing the symbiotic program at the transcriptional level (Das et al., 2022). MYCS represents another class of conserved elements found in the promoters of AM-related genes. Comparative analysis demonstrates that MYCS elements are widely present in the promoters of AM-specific transporter genes in Solanaceae and Fabaceae species, yet they have not been identified in rice. The deletion of either the P1BS or the MYCS element abolishes the AM-specific activity of the promoter, suggesting that these two classes of elements act synergistically to achieve full symbiosis-specific transcriptional activation. This dual-element dependency implies that AM-specific expression requires the cooperative assembly of multiple transcriptional regulators (Xu et al., 2023**;** Ansari et al., 2026**;** Chen et al., 2011). In *Medicago truncatula*, WRI5a directly binds to the AW-box within target gene promoters to regulate the expression of genes involved in fatty acid biosynthesis and phosphate transport; meanwhile, in *Lotus japonicus*, CBX1 recognizes the CTTC motif to perform a similar function. These elements are primarily involved in gene regulation during the functional maturation stage of arbuscules. Recent studies have also revealed that WRI5a binds to the AW-box (located at -396 to -420 bp) in the promoter of an auxin efflux transporter *ABCB1* and activates its expression to promote arbuscule maintenance, thereby broadening our understanding of the functional scope of AM promoter regulatory networks (Ho-Plágaro and García-Garrido, 2022**;** Wang et al., 2026). A novel motif has also been identified in the promoters of *MtLec5* and *MtLec7*. By comparing these two co-regulated AM-specific lectin genes, researchers identified a conserved 10-bp motif (AACATTTTGT). Located on the sense and antisense strands of the respective promoters and showing no homology to any known regulatory elements, this motif may represent a novel class of binding sites for AM-specific transcription factors. Interestingly, this motif was not found in the promoters of other known AM-specific genes, suggesting that AM genes belonging to different functional categories may be driven by distinct regulatory modules (Chen et al., 2011**;** Frenzel et al., 2005**;** Frenzel et al., 2006). GRAS-family transcription factors occupy key positions within the transcriptional regulatory network of AM symbiosis. The complex formed by CYCLOPS and DELLA proteins directly regulates the expression of *RAM1*; acting as a key upstream regulator, RAM1 not only activates genes associated with arbuscule maintenance but also participates in a positive regulatory loop with WRI5a, in which the two factors reinforce the transcriptional program required for arbuscule maturation. Furthermore, NSP1 and NSP2 regulate the expression of genes involved in SL biosynthesis, while the DELLA-MYB1 module participates in regulating genes associated with arbuscule degeneration. This multi-layered transcriptional cascade suggests that the activation of AM-specific promoters results from the sequential or cooperative action of multiple transcription factors (Ho-Plágaro and García-Garrido, 2022**;** Pimprikar et al., 2016**;** Yu et al., 2014**;** Floss et al., 2017).

Although several AM-specific cis-elements have been identified, the field still faces several core challenges. First, reliably predicting conserved regulatory motifs from a limited number of known promoter sequences remains a bioinformatics challenge. Second, compared to the relatively well-defined organ-specific elements (OSEs) found in root nodule symbiosis, the regulation of AM-specific genes appears more complex, involving the combinatorial action of multiple promoter modules. In the future, integrating high-throughput promoter activity screening and gene-editing technologies holds the promise of systematically mapping the regulatory landscape of AM-specific promoters, thereby providing molecular tools for the precise manipulation of symbiotic benefits (**Table 2**).

**Table 2:** Regulatory network controlling PT4/PT11 expression during arbuscular mycorrhizal symbiosis.

Regulatory level	Representative regulator(s)	Mode of regulation	Target/Mechanism	Effect on PT4/PT11	Representative references
Plasma membrane receptors	SYMRK/DMI2, CERK1 homologs	Signal transduction	Initiate the common symbiosis signaling pathway (CSSP) through receptor-mediated perception of Myc factors	Positive	(Antolín-Llovera et al., 2014)
Nuclear calcium signaling	CASTOR, POLLUX, NUP85, NUP133	Nuclear Ca²^+^ oscillation	Generate nuclear calcium spiking required for downstream signaling	Positive	(Charpentier et al., 2016)
Calcium decoder	CCaMK	Protein kinase	Decodes Ca²^+^ signatures and phosphorylates CYCLOPS	Strong positive	(Singh and Parniske, 2012)
Central transcriptional regulator	CYCLOPS	Transcriptional activation	Directly activates RAM1 transcription	Strong positive	(Pimprikar et al., 2016)
Master regulator	RAM1 (GRAS TF)	Direct transcriptional activation	Activates AM-associated transcriptional programs, including phosphate transport and arbuscule-development genes	Essential positive regulator	(Gobbato et al., 2012)
AP2/ERF transcription factor	WRI5a	Direct activation	Coordinates lipid metabolism and phosphate transporter expression	Positive	(Jiang et al., 2017; Guo et al., 2022)
AP2/ERF transcription factor	CBX1	Direct transcriptional activation	Activates PT11 together with lipid-transfer genes	Positive	(Xue et al., 2018)
GRAS proteins	DELLA proteins	Protein interaction	Form complexes with CYCLOPS/RAM1 to enhance transcription	Positive	(Floss et al., 2013)
Additional transcription factors	RAD1, MIG1, NSP1/NSP2	Transcriptional regulation	Coordinate cortical cell differentiation and AM development	Positive	(Park et al., 2015; Liu et al., 2011)
Hormonal regulation	Strigolactones	Hormone signaling	Promote fungal branching and PT4/PT11 induction	Positive	(Akiyama et al., 2005)
	Abscisic acid	Hormone signaling	Facilitates AM establishment and transporter expression	Positive	(Herrera-Medina et al., 2007)
	Gibberellins	DELLA degradation	Destabilizes DELLA proteins and suppresses RAM1 pathway	Negative	(Floss et al., 2013)
	Ethylene	Hormone signaling	Restricts AM colonization under some conditions	Mostly negative	(Foo et al., 2013)
Phosphate signaling	PHR1/PHR2	Pi starvation response	Coordinates systemic phosphate starvation signaling	Positive under low Pi	(Bari et al., 2006)
	SPX proteins	Pi sensor	Inhibit PHR activity under Pi sufficiency	Negative	(Puga et al., 2014)
	miR399–PHO2 module	Systemic Pi signaling	Modulates phosphate homeostasis and transporter abundance	Indirect	(Lin et al., 2008)
Kinase signaling module	KINASE3 (KIN3), AMK8, AMK24, OsRLCK171	Receptor-like kinase cascade	CBX1 directly activates KIN3; AMK8/24 interact with KIN3 to regulate arbuscule development and nutrient exchange	Indirect positive	Leng et al., 2023
Negative-feedback regulation	ERM1–ERF12–TPL module	Transcriptional repression	WRI5a activates ERF12, which recruits TPL to attenuate excessive arbuscule development and indirectly fine-tune PT4/PT11 activity	Fine-tuning / Negative feedback	Zhang et al., 2023
Emerging regulatory mechanisms	Small molecules, single-cell transcriptomics, spatial transcriptomics	Multi-omics regulation	Cell-type-specific regulatory networks reveal additional regulators controlling arbuscule-containing cortical cells and PT4/PT11 expression	Putative positive regulators	Delaux and Gutjahr, 2024
Environmental regulation	External Pi availability	Nutrient feedback	High Pi suppresses AM development and PT4/PT11 expression	Negative	(Smith and Smith, 2011)

## 3 Regulation of PT4 and PT11 expression during AM symbiosis

The establishment of the AM symbiosis begins with a series of intracellular signal transduction events triggered by the host root system’s perception of fungal signaling molecules, the conserved signaling module known as CSSP. Given that this module plays a critical role in both AM symbiosis and the legume-rhizobium symbiosis, it suggests that the signaling mechanism of the latter evolved from the more ancient AM symbiotic pathway (Paszkowski et al., 2006**;** Stracke et al., 2002**;** Díaz et al., 2025**;** Genre and Russo, 2016).

### 3.1 Transcriptional regulation

Upon CSSP activation, downstream transcriptional regulatory networks converge on promoters of symbiosis-associated genes such as *PT4* and *PT11*, contributing to their expression in arbuscule-containing cortical cells. This transcriptional regulatory network exhibits a multi-tiered structural organization, involving the cooperative action of multiple GRAS-family transcription factors and the combinatorial encoding of cis-regulatory elements (Gutjahr et al., 2008**;** Yang et al., 2022**;** Das et al., 2022). Downstream of the CSSP, the RAM1 transcription factor was the first to be definitively identified as a key regulator directly controlling the expression of *PT4* and *PT11*. RAM1 belongs to the GRAS family of transcription factors, and its expression is co-activated by the CCaMK-CYCLOPS complex and DELLA proteins. In *ram1* mutants, although AM fungi can colonize the root system, arbuscule maintenance is aberrant and *PT4* expression is drastically reduced; this suggests that RAM1 serves as a key functional component linking early calcium signaling with the expression of genes involved in nutrient exchange. Two additional transcription factors, WRI5a in *M. truncatula* and CBX1 in *L. japonicus*, are members of the AP2/ERF family and regulate overlapping aspects of arbuscule maintenance and nutrient exchange. Both have been implicated in the regulation of *PT4*. *WRI5a* is specifically expressed during arbuscule formation. The *wri5a* mutant exhibits impaired arbuscule maturation and significantly reduced *PT4* expression. Notably, RAM1 and WRI5a physically interact and cooperatively regulate downstream genes, thereby reinforcing the transcriptional program associated with arbuscule maturation and nutrient exchange. This interaction may contribute to sustained expression of symbiosis-associated genes during arbuscule maturation. Furthermore, WRI5a regulates the expression of genes involved in fatty acid biosynthesis, thereby coordinating phosphate transport with carbon source supply at the transcriptional level (Pimprikar et al., 2016**;** Paries et al., 2025**;** Zhang et al., 2023).

The regulation of *PT4*/*PT11* by transcription factors relies on the recognition of specific cis-elements within their promoters. Several functional cis-elements have been identified in the promoters of AM- specific phosphate transporter genes, including *MtPT4* and *OsPT11*: the *P1BS* element serves as the binding target for PHR transcription factors, which respond to phosphate status. PHRs are activated under low phosphate conditions and upregulate the expression of a suite of phosphate-starvation-induced genes by binding to *P1BS*. While the presence of P1BS confers a basal response to phosphate status, it alone is insufficient to drive arbuscule-specific expression. The AW-box acts as the binding site for WRI5a, an AP2/ERF-family transcription factor; WRI5a directly activates *PT4* transcription by binding to the AW-box within its promoter. Functional studies suggest that P1BS and AW-box-containing regulatory modules contribute substantially to AM-specific promoter activity, as the mutation or deletion of either element severely impairs the AM-specific activity of the promoter.

Functional studies indicate that P1BS and AW-box-containing regulatory modules contribute substantially to AM-specific promoter activity. PHR-dependent regulation through *P1BS* links promoter activity to phosphate status, whereas WRI5a binding to the AW-box contributes to enhanced expression in arbuscule-containing cells. Mutational analyses of these elements have demonstrated substantial reductions in promoter activity, supporting a model in which multiple cis-regulatory modules cooperate to achieve appropriate spatial and developmental regulation of AM-specific phosphate transporter expression. However, the relative contribution and potential combinatorial interactions of these regulatory elements may differ among plant species and transporter genes (Pimprikar et al., 2016**;** Chakravarthy et al., 2003**;** Paries et al., 2025).

In summary, the transcriptional regulation of PT4/PT11 constitutes a complex network involving the synergistic action of multiple factors across interconnected regulatory layers. From upstream CSSP-dependent transcriptional activation involving RAM1 and WRI5a, through the combinatorial coding of P1BS and AW-box elements at the promoter level, to SPX-mediated negative feedback regulation by phosphate status, this network ensures the precise spatiotemporal expression of phosphate transporter genes, serving as the molecular basis for efficient nutrient exchange in AM symbiosis. Future elucidation of this regulatory pathway in non-model crops will provide potential molecular targets for optimizing symbiotic benefits through transcriptional regulation.

### 3.2 Hormonal regulation of AM symbiosis and implications for PT4/PT11

Plant hormones are endogenous trace organic molecules that serve as critical intercellular signals. At extremely low concentrations, they govern virtually all phases of the plant life cycle, encompassing germination, vegetative growth, reproductive development, and senescence, and concurrently enable plants to cope with abiotic stresses including drought and chilling stress (Ma et al., 2026; Murtaza et al., 2026).

Beyond the fine-tuned control exerted by the CSSP and downstream transcriptional regulatory networks on PT4/PT11, the multifaceted roles played by phytohormones represent a regulatory dimension that cannot be overlooked. Plants employ virtually all major classes of phytohormones to modulate their relationships with AM fungi. These hormonal signals interact with nutritional status, developmental programs, and transcriptional networks to form a complex regulatory system. Consequently, this section focuses on how various phytohormones directly or indirectly influence *PT4*/*PT11* expression and symbiotic phosphate transport function.

#### 3.2.1 Regulatory roles of auxin and cytokinin

Auxin (IAA) plays a key regulatory role in the symbiotic relationship between plants and AM fungi; numerous studies have confirmed that IAA acts as a positive signal that promotes and finely regulates this symbiosis. AM fungal colonization significantly increases IAA levels in plant roots, which can promote root architectural changes, including increased lateral root and root hair formation. Auxin signaling may also directly contribute to arbuscule formation (Chen et al., 2022**;** Liu et al., 2024a**;** Etemadi et al., 2014). Fitze et al. determined the levels of free, esterified, and amide-conjugated IAA in maize roots inoculated with *Glomus intraradices* during the early stages of arbuscular mycorrhiza formation. They found that the levels of amide conjugates of IAA and indole-3-butyric acid (IBA) increased significantly at some days post-inoculation. The production of free and conjugated IBA was systemically induced during arbuscular mycorrhizal colonization of maize leaves. This suggests that the synthesis and hydrolysis of auxin conjugates play a crucial role in the formation of arbuscular mycorrhizae. Consequently, their results suggest the existence of complex regulatory mechanisms operating at both local and systemic levels that modulate the levels of free and conjugated auxins during the early stages of arbuscular mycorrhizal symbiosis (Fitze et al., 2005).

Cytokinin (CK) regulation of PT4/PT11 transporters exhibits intriguing organ-specific differentiation. Studies using transgenic tobacco have revealed a sophisticated dual-regulatory model: shoot-derived CK positively influences intraradical AM fungal development and the transcriptional levels of the AM-responsive phosphate transporter gene *NtPT4*; conversely, root-derived CK appears to act as a brake, limiting the supply of carbon sources from the root to the fungus to prevent the symbiotic relationship from shifting into parasitism. Although CK levels generally increase in AM-colonized plants, their function is not merely one of unidirectional promotion; rather, they perform distinct regulatory tasks across different tissue levels, suggesting that plants may precisely balance nutrient investment and returns within the symbiosis through the compartmentalized distribution of CK (Großkinsky et al., 2025**;** Liao et al., 2018).

#### 3.2.2 Regulatory networks involving gibberellins, abscisic acid, and jasmonic acid

Similar to the multifaceted roles of cytokinins (CK), gibberellins (GA), abscisic acid (ABA), and jasmonic acid (JA) exhibit distinct and context-dependent effects on arbuscular mycorrhizal (AM) symbiosis. GA generally acts as a negative regulator of arbuscule formation by promoting the degradation of DELLA proteins, which are required for efficient arbuscule maintenance. DELLA proteins interact with components of the common symbiosis signaling pathway, including *CYCLOPS*, and contribute to the activation of *RAM1* expression. Thus, GA signaling can modulate the transcriptional program associated with arbuscule maintenance through the GA–DELLA–CYCLOPS–RAM1 regulatory module. Because RAM1 regulates multiple genes involved in arbuscule maintenance and nutrient exchange, including AM-associated phosphate transporter genes, changes in GA–DELLA signaling may ultimately influence *PT4*/*PT11* expression indirectly (Floss et al., 2013**;** Pimprikar et al., 2016). In contrast, ABA has been reported to positively influence aspects of AM symbiosis, although its effects may depend on developmental stage, hormone concentration, and environmental context. Previous studies have also shown that ABA and JA can modulate AM symbiosis and may indirectly affect *PT4*/*PT11* expression through their effects on symbiotic development, hormone crosstalk, and host physiological status (Floss et al., 2013**;** Xue et al., 2015). JA, in particular, exhibits context-dependent effects on AM symbiosis, with its influence varying according to hormone concentration, developmental stage, host genotype, and environmental conditions. JA may affect symbiotic interactions by modulating carbon allocation to colonized roots and/or by altering host defense responses. However, the molecular mechanisms linking ABA or JA signaling specifically to PT4/PT11 transcription remain insufficiently resolved. Plant hormone signaling pathways do not operate independently but interact extensively with the transcriptional networks that regulate AM symbiosis. DELLA proteins provide a well-characterized example of such integration. As key targets of GA signaling, DELLA proteins interact with CYCLOPS and contribute to the activation of *RAM1* expression, thereby connecting hormonal status with the symbiotic transcriptional program. GA-induced DELLA degradation can consequently attenuate this regulatory module and impair arbuscule maintenance. However, the extent to which these hormonal effects are transmitted specifically to *PT4*/*PT11* expression may vary among plant species and developmental contexts and should not be interpreted as evidence for a direct hormonal regulation of PT4/PT11.

Other hormonal pathways may also intersect with phosphate and symbiosis signaling. Auxin signaling interacts with strigolactone (SL) biosynthesis and signaling, while PHR-dependent phosphate signaling has been implicated in the regulation of SL biosynthesis, providing a potential connection between plant phosphate status and the early stages of AM symbiosis (Park et al., 2013**;** Lei et al., 2020**;** Thilakarathne et al., 2025). These interactions further indicate that the regulation of AM-associated phosphate transport occurs within a broader network integrating hormonal, nutritional, and symbiotic signals.

Overall, hormonal regulation of PT4/PT11 and their upstream transcriptional networks is best viewed as a multilayered and context-dependent process involving interconnected positive and negative regulatory pathways. The GA–DELLA module provides a well-supported example of how hormone signaling can intersect with the AM transcriptional program, whereas the effects of CK, ABA, JA, auxin, and SL are likely to depend on developmental and nutritional contexts. Rather than establishing a single linear hormonal pathway controlling PT4/PT11, current evidence supports a model in which hormonal and nutritional signals converge on symbiosis-associated transcriptional networks and thereby contribute to the appropriate regulation of phosphate transporter expression during AM symbiosis.

### 3.3 Nutrient feedback with AM symbiosis

In the preceding sections, we explored how CSSP initiates symbiotic signaling, how transcription factor networks drive the expression of PT4/PT11, and how phytohormones regulate this process across multiple dimensions. However, these regulatory mechanisms do not operate in a vacuum; they are subject to continuous monitoring and dynamic adjustment based on the plant’s internal nutritional status. This section focuses on how nutritional feedback acts as an economic valve, determining based on the host’s phosphorus demand whether to maintain, enhance, or suppress the investment in phosphate transport within the AM symbiosis (Shi et al., 2021**;** Aizaz et al., 2024**;** Das et al., 2022).

#### 3.3.1 Systemic feedback on phosphate nutritional status

The expression of *PT4*/*PT11* is highly sensitive to internal phosphate levels, a feedback response mediated primarily by the PHR-SPX regulatory module. SPX proteins negatively regulate PHR activity in response to cellular phosphate and inositol pyrophosphate status. The SPX protein itself lacks the ability to directly bind to the PHR transcription factor; its function depends on the intracellular presence of InsP8. The intracellular concentration of InsP8 is positively correlated with the level of P_i_ availability—when plants are under P_i_-sufficient conditions, InsP8 accumulates in large quantities; This small molecule, acting as the actual phosphate status signal, is specifically recognized and bound by the SPX domain of the SPX protein. Upon binding to InsP8, SPX undergoes a conformational change, exposing an interface that interacts with PHR, thereby stably recruiting and inhibiting PHR’s transcriptional activation activity, which shuts down the expression of phosphate starvation response genes. Conversely, under P_i_-deficient conditions, the concentration of InsP8 drops sharply; SPX is unable to bind this ligand and therefore cannot interact with PHR, allowing PHR to freely activate the starvation response program. Thus, SPX–PHR association is strongly dependent on InsP8 binding, supporting a role for InsP8 as a key molecular indicator of intracellular phosphate status (Wild et al., 2016**;** Lv et al., 2014**;** Dong et al., 2019).

Under phosphate starvation, PHR proteins bind to P1BS elements in the promoters of target genes, thereby activating phosphate-starvation-induced genes, including *PT4/PT11*. Conversely, when internal phosphate levels are sufficient, SPX proteins (SYG1/PHO81/XPR1) interact directly with PHRs, inhibiting their DNA-binding activity and/or nuclear localization, which blocks P1BS-mediated transcriptional activation. Given that P1BS is a critical component of the AM-responsive promoter architecture, along with AW-box and MYCS elements, this feedback mechanism effectively suppresses the expression of symbiotic phosphate transporter genes under high-phosphate conditions. When a plant can already obtain sufficient phosphate from the soil via its own root system, the investment of continuing to supply carbon sources to AM fungi in exchange for additional phosphate is economically disadvantageous (Ge et al., 2026**;** Sega and Pacak, 2019**;** Lin et al., 2025). Thus, suppression of *PT4*/*PT11* under high-phosphate conditions may represent a mechanism by which plants reduce investment in the mycorrhizal phosphate uptake pathway when the nutritional benefit of maintaining the symbiosis is reduced.

#### 3.3.2 Feedback regulation of carbon source supply

The balance between phosphate acquisition and carbon expenditure lies at the heart of the economics of AM symbiosis. As discussed previously, under high-phosphate conditions, plants do not require AM fungi for nutrient uptake; consequently, they restrict the flow of carbon to the fungi to minimize carbon loss. There is an intrinsic coupling between the transfer of photosynthetic carbon, including sugars and fatty acids, from the plant to the fungus and the phosphate returned by the fungus. Experimental evidence shows that when photosynthesis is inhibited through shading treatments, the expression levels of *PT4* and *PT11* decline, leading to reduced efficiency in symbiotic phosphate uptake (Konečný et al., 2019). This observation is consistent with the possibility that phosphate transport capacity is adjusted in response to carbon availability, although the molecular mechanisms connecting carbon status, symbiotic carbon allocation, and *PT4*/*PT11* expression remain incompletely understood. (Ryan et al., 2012**;** Konečný et al., 2019**;** Balzergue et al., 2013). Plants integrate carbon status with the expression of phosphate transport genes via the RAM1-WRI5a positive feedback loop. WRI5a regulates not only *PT4*/*PT11* but also directly controls the expression of genes involved in fatty acid biosynthesis, thereby coupling phosphate acquisition capacity (PT4/PT11) with carbon payment capacity (fatty acid synthesis) at the transcriptional level. When the supply of photosynthetic products is insufficient, this loop may be interrupted by an energy signal not yet fully elucidated leading to the coordinated downregulation of both the maintenance costs of arbuscules and the benefits of phosphate uptake (Paries et al., 2025**;** Shi et al., 2021**;** Zhang et al., 2023).

#### 3.3.3 On the regulation of AM symbiosis by nitrogen and zinc

Although phosphate is the primary nutrient exchanged in AM symbiosis, the status of other nutrients—such as nitrogen (N) and zinc (Zn) also influences the expression of *PT4*/*PT11*. While nitrogen sufficiency can promote AM symbiosis, studies have also shown that it can inhibit the process; thus, the impact of nitrogen on AM symbiosis is multifaceted and remains to be fully elucidated. This N feedback may act on upstream regulators of PT4/PT11 via independent signaling pathways or indirectly influence carbon allocation by altering the plant’s overall carbon-to-nitrogen balance, thereby modulating investment in the symbiosis. Similarly, soil zinc availability affects the efficiency of AM symbiosis establishment, though it remains unclear whether this feedback directly regulates PT4/PT11 transcription (Rui et al., 2022**;** Xie et al., 2019**;** Nouri et al., 2014).

#### 3.3.4 Dynamic feedback of phosphate transport and the arbuscule life cycle

The expression of PT4/PT11 changes dynamically during the arbuscule life cycle, generally increasing during arbuscule maturation and declining as arbuscules undergo senescence. This temporal pattern is consistent with their role in phosphate acquisition at the mature symbiotic interface. Changes in cellular phosphate status may also contribute to the regulation of PT4/PT11 expression through the PHR–SPX regulatory system. However, the extent to which phosphate taken up specifically through PT4/PT11 contributes to this feedback remains unresolved. Thus, the decline in PT4/PT11 expression during arbuscule senescence should not be interpreted solely as a direct consequence of transporter-mediated phosphate accumulation, because transcriptional repression associated with developmental progression and other nutrient- and hormone-dependent mechanisms may also contribute (Yang et al., 2012**;** Luginbuehl and Oldroyd, 2017**;** Kobae, 2019).

In summary, situating nutrient feedback within the multi-level regulatory framework established in the preceding sections clearly demonstrates its structural role. The upstream CSSP module acts as a switch, determining whether to initiate the symbiotic program; the intermediate transcription factor network (RAM1-WRI5a) functions as an amplifier, driving high-level expression of *PT4*/*PT11*, fine-tuning output intensity based on the plant’s cost-benefit calculations. Current and future research aims to elucidate how nutrient signals precisely regulate this transcriptional network at the molecular level, thereby identifying targets for sustainable agricultural strategies that optimize symbiotic benefits and reduce reliance on chemical fertilizers through genetic improvement.

### 3.4 Post-translational regulation and trafficking of PT4/PT11

Although transcriptional regulation is a major determinant of PT4/PT11 abundance during AM symbiosis, the accumulation of functional transporters at the PAM also requires post-translational control of protein trafficking and turnover. As members of the PHT1 phosphate transporter family, PT4/PT11 must undergo proper intracellular trafficking before reaching their functional membrane domain. Studies of non-mycorrhizal PHT1 transporters have demonstrated that the phosphate transporter traffic facilitator 1 (PHF1) promotes their exit from the endoplasmic reticulum, while phosphorylation of PHT1 proteins can modulate ER export and subsequent plasma-membrane localization (Bayle et al., 2011**;** González et al., 2005). These findings indicate that phosphate transporter abundance at the target membrane is controlled not only transcriptionally but also through post-translational mechanisms affecting protein localization and stability.

In AM symbiosis, PT4 and PT11 are specifically accumulated at the PAM surrounding arbuscule branches, indicating that selective membrane targeting is an essential component of their function. Recent live-cell imaging of OsPT11 further revealed dynamic changes in its abundance and distribution during arbuscule development. PT11 was consistently associated with the PAM of fine arbuscule branches, whereas its abundance changed according to arbuscule developmental stage and phosphate status (McGaley et al., 2026). These observations suggest that the amount of functional PT11 at the PAM is dynamically regulated and may involve post-translational mechanisms in addition to transcriptional control. However, the molecular factors that specifically mediate PT4/PT11 trafficking to, retention at, or removal from the PAM remain largely unknown.

Post-translational regulation of PHT1 proteins can also involve endocytosis, recycling, ubiquitination, and vacuolar degradation. In Arabidopsis, PHT1;1 undergoes internalization from the plasma membrane and can either be recycled under phosphate-limited conditions or targeted for vacuolar degradation when phosphate is abundant (Bayle et al., 2011). In addition, the E3 ubiquitin ligase NITROGEN LIMITATION ADAPTATION (NLA) promotes ubiquitination, clathrin-dependent endocytosis, and vacuolar degradation of plasma-membrane-localized PHT1 proteins, while the PHO2/UBC24 pathway contributes to the regulation of PHT1 abundance in intracellular compartments (Lin et al., 2013**;** Huang et al., 2013). These mechanisms provide a potential framework for understanding how phosphate transporter abundance can be rapidly adjusted at the membrane. Nevertheless, whether PT4/PT11 are regulated by the same ubiquitination, endocytic, and recycling mechanisms during AM symbiosis remains unclear. In particular, direct evidence for PT4/PT11-specific phosphorylation sites, ubiquitination events, recycling from the PAM, or AM-specific trafficking factors is currently limited.

Thus, post-translational regulation of PT4/PT11 represents an important but still emerging layer of control. Future studies integrating live-cell imaging, phosphoproteomics, ubiquitinome analysis, proximity labeling, and genetic approaches will be valuable for identifying the mechanisms controlling PT4/PT11 delivery, retention, recycling, and degradation at the PAM. Such studies may clarify how plants dynamically adjust phosphate uptake capacity during different stages of arbuscule development and under changing nutritional conditions.

## 4 Functional coupling of PT4/PT11-mediated phosphate transport with arbuscule maintenance

The preceding sections describe multiple layers of PT4/PT11 regulation, ranging from common symbiosis signaling and transcriptional activation to hormonal and nutritional control and, potentially, post-translational regulation of protein trafficking and turnover. An important question is how these regulatory processes relate to the maintenance of functional arbuscules. Genetic studies have consistently shown that impairment of PT4/PT11-mediated phosphate uptake is associated with defective phosphate acquisition and premature arbuscule degeneration. These observations establish a strong functional connection between symbiotic phosphate transport and arbuscule maintenance. However, they do not by themselves demonstrate that PT4/PT11 directly regulate developmental or signaling pathways independently of their transport activity.

Current evidence therefore supports a transport-dependent model in which phosphate uptake through PT4/PT11 contributes to the physiological conditions required for sustained nutrient exchange and arbuscule function. At the protein level, regulation of transporter delivery, retention, recycling, and degradation could additionally determine the amount of functional PT4/PT11 available at the PAM and thereby modulate phosphate uptake capacity. Whether such post-translational mechanisms contribute directly to the developmental transitions of arbuscules remains unresolved. In the following sections, we distinguish experimentally supported observations from mechanistic models that require further validation.

### 4.1 PT4/PT11 and arbuscule development

Arbuscule development requires extensive remodeling of the host cortical cell and the formation of a highly specialized PAM. This process depends on polarized vesicle trafficking and targeted exocytosis to deliver membrane components and symbiosis-associated proteins to the developing arbuscule. Live-cell imaging and fluorescent marker studies have provided evidence for dynamic remodeling of endomembrane and cytoskeletal systems during arbuscule formation (Ivanov and Harrison, 2014). Importantly, the AM-induced exocyst subunit EXO70I is specifically recruited to developing arbuscule branches, and loss of EXO70I impairs arbuscule branching and the incorporation of PAM-localized transport proteins, indicating that exocyst-mediated secretion contributes directly to PAM biogenesis and arbuscule maturation (Zhang et al., 2015). Together with the functions of symbiosis-associated SNARE proteins, these findings indicate that vesicle trafficking is an essential cellular process underlying the establishment and functional specialization of the arbuscular interface. Symbiosis-associated SNARE proteins, including MtVAMP721d/e and SYP132α, further contribute to the delivery and fusion of vesicles required for proper arbuscule development (Huisman et al., 2020).

### 4.2 Arbuscule maturation

#### 4.2.1 Spatiotemporal coupling between PT4/PT11 expression and the maturation process of arbuscules

Arbuscule maintenance is a dynamic, continuous process that can be broadly divided into four stages: invasion, branching/expansion, maturation, and senescence. During the invasion and early branching stages, the expression of *PT4*/*PT11* remains at a low level or has not yet been initiated. As the arbuscular branching network progressively expands within the cortical cells, PT4/PT11 transcription is rapidly induced, peaking during the maturation stage; the resulting proteins are precisely localized to the PAM—the critical interface where substantial nutrient exchange between the plant and fungus occurs (Floss et al., 2013**;** Alexander et al., 1989**;** Zhang et al., 2023). Initiating high-level phosphate transport before arbuscular branches have fully expanded is energetically inefficient, as the fungus has not yet established a sufficiently large interface area for phosphate release; conversely, once the arbuscules mature and the fungal partner begins releasing phosphate in large quantities, the plant must immediately possess efficient phosphate uptake capabilities to prevent the loss of phosphate from the interfacial space. Therefore, the upregulation of PT4/PT11 is not merely a consequence of arbuscular maturation but serves as a marker of its completion (Balzergue et al., 2013**;** Kobae, 2019**;** Harrison et al., 2002).

#### 4.2.2 Effect of PT4/PT11 deletion on arbuscule maturation: a phenotype of premature senescence

The most significant impact of PT4/PT11 transport dysfunction on arbuscule maintenance is the loss of the ability to maintain the mature state. In *Medicago truncatula*, the *Mtpt4* mutant can still be successfully colonized by AM fungi and can form morphologically intact arbuscules; however, these arbuscules undergo premature degeneration shortly after formation. Unlike wild-type arbuscules, which maintain a mature phase lasting several days, those in *mtpt4* mutants typically begin to collapse within 1–2 days of formation, manifesting as branch shrinkage, membrane disintegration, and condensation of the fungal cytoplasm before rapidly entering senescence. The mature phase of an arbuscule is not merely a passive window of time preceding senescence, but an active state that requires continuous functional input for its maintenance. PT4/PT11-mediated phosphate transport represents one such essential functional input. In the absence of this transport activity, arbuscules may be born, but they fail to survive long enough to fulfill their role in nutrient delivery (Javot et al., 2011**;** Harrison et al., 2002).

#### 4.2.3 Potential consequences of altered interfacial phosphate availability for arbuscule longevity

How might PT4/PT11-mediated phosphate transport be functionally connected to arbuscule longevity? One possibility is that changes in transporter activity alter phosphate availability and other physiological properties of the plant–fungus interface. During normal symbiosis, phosphate released by the fungus into the PAS is taken up by the plant through PT4/PT11. Efficient phosphate uptake is therefore expected to influence phosphate flux across the interface and may contribute to maintaining the physiological conditions required for sustained nutrient exchange. When PT4/PT11 function is impaired, phosphate uptake from the PAS is reduced. This may alter local phosphate availability and the balance of nutrient exchange between the two symbiotic partners. Such changes could affect fungal metabolism, host nutrient status, carbon allocation, or other processes associated with the maintenance of mature arbuscules. However, whether interfacial phosphate accumulation itself is directly sensed as a signal by the host or fungus has not yet been established. Similarly, there is currently no direct evidence that PT4/PT11 transmit an intracellular signal independently of their transport activity (Volpe et al., 2016**;** Gu et al., 2011**;** Javot et al., 2007b).

Morphological and ultrastructural abnormalities observed in *PT4*-deficient roots, including alterations at the plant–fungus interface, are consistent with the possibility that impaired phosphate transport affects the physiological environment required for stable arbuscule maintenance. Nevertheless, these observations do not distinguish between direct effects of altered phosphate flux and secondary consequences arising from changes in carbon allocation, fungal metabolism, or host nutrient status. Thus, the relationship between PT4/PT11-mediated phosphate transport and arbuscule longevity should currently be regarded as a transport-dependent functional association rather than an established signaling mechanism. Future experiments combining quantitative measurements of interfacial phosphate concentrations, phosphate flux, carbon allocation, and arbuscule maintenance states will be required to determine how changes in PT4/PT11 activity are translated into changes in arbuscule fate (Volpe et al., 2016**;** Gu et al., 2011**;** Javot et al., 2007b).

In summary, PT4 and PT11 are essential components of the mycorrhizal phosphate uptake pathway, and their transport activity is closely associated with the maintenance of mature arbuscules. Loss of *PT4*/*PT11* function impairs phosphate uptake and is accompanied by premature arbuscule degeneration. These observations support a strong functional relationship between phosphate transport and arbuscule maintenance, but they do not establish PT4/PT11 as direct regulators of arbuscule maintenance signaling. A more conservative interpretation is that PT4/PT11-mediated phosphate transport contributes to the physiological and nutritional conditions required for sustained arbuscule function. The molecular processes connecting altered phosphate flux with arbuscule degeneration remain to be determined.

### 4.2 Arbuscule degeneration

Arbuscule degeneration represents the final stage of the AM symbiosis lifecycle, marking the termination of the symbiotic relationship between the fungus and the individual cortical cell. This process involves coordinated cellular and molecular changes in both the host and fungal partners and is accompanied by active clearance of fungal structures by the host cell. Complementing the discussion in the previous section regarding the positive roles of PT4 and PT11 in maintaining arbuscule maturity, this section focuses on the triggering mechanisms and molecular markers of arbuscule degeneration, as well as the underlying mechanisms by which the loss of *PT4*/*PT11* function accelerates this process.

#### 4.2.1 Morphological and cytological characteristics of arbuscule senescence

Arbuscule senescence is a clearly identifiable degenerative process. At the cellular level, senescing arbuscules exhibit the contraction and collapse of their tree-like branching structures, a reduction in branch lumen diameter, and fungal cytoplasmic condensation and vacuolization, ultimately leading to organelle degradation and cellular collapse. Concurrently, the plant-derived PAM gradually loses the structural integrity of its close apposition to the fungal branches; the membrane surface area decreases, and the interfacial gap widens. This degenerative process is accompanied by heightened lysosomal/vacuolar activity within the plant cell, indicating that the plant cell actively participates in the clearance of arbuscules. Within the same root segment or even among adjacent cortical cells, one often observes the coexistence of arbuscules at various developmental stages, ranging from newly formed and functionally mature arbuscules to those undergoing senescence (Park et al., 2015**;** Toth and Miller, 1984**;** Carrión et al., 2014). Thus, the coexistence of arbuscules at different developmental stages within the same root segment is consistent with substantial local or cell-associated regulation of arbuscule senescence. However, the contribution of systemic signals cannot be excluded, and the relative importance of local versus systemic regulatory mechanisms remains to be determined.

#### 4.2.2 Molecular mechanisms underlying PT4/PT11 loss of function and accelerated arbuscule senescence

In *Mtpt4* and *Ospt11* mutants, arbuscules undergo premature degeneration following their formation, resulting in a shortened period of functional symbiosis. This phenotype raises an important question concerning how impaired PT4/PT11-mediated phosphate transport is associated with arbuscule senescence. Several, not mutually exclusive, mechanisms may contribute to this relationship. One possibility is that reduced phosphate uptake alters the physicochemical and nutritional conditions of the plant–fungus interface. Under normal symbiosis, phosphate released by the fungus into the peri-arbuscular space is taken up by the host through PT4/PT11. Impairment of this transport activity would therefore be expected to alter phosphate flux across the interface and could modify local nutrient availability. However, the extent to which phosphate accumulates specifically within the peri-arbuscular space in PT4/PT11-deficient cells, and whether such changes are directly sensed to initiate arbuscule degeneration, remain to be experimentally determined (Javot et al., 2007a**;** Javot et al., 2007b**;** Javot et al., 2011**;** Choi et al., 2018**;** Jia et al., 2025).

A second possibility is that impaired phosphate uptake alters the overall nutritional balance of the symbiotic interaction. Because AM symbiosis involves reciprocal exchange of fungal-derived phosphate for host-derived carbon and lipids, disruption of phosphate transfer may affect fungal metabolism, host phosphate status, carbon allocation, or other processes required for sustained arbuscule function. These effects could contribute to the premature degeneration observed in transporter-deficient mutants, but the relative contribution of each mechanism remains unresolved. Thus, the accelerated senescence observed following *PT4*/*PT11* loss should currently be interpreted as a strong functional association between symbiotic phosphate transport and arbuscule maintenance rather than as evidence for a defined phosphate-sensing pathway downstream of PT4/PT11(Zhang et al., 2016**;** Chiu and Paszkowski, 2019**;** Dreyer et al., 2019**;** Zhang et al., 2016**;** Chiu and Paszkowski, 2019; Dreyer et al., 2019**;** Dindas et al., 2022**;** Li et al., 2025**;** Agisha et al., 2024). Future studies combining quantitative measurements of phosphate dynamics at the plant–fungus interface with analyses of carbon allocation, fungal metabolism, and arbuscule maintenance states will be necessary to determine how impaired phosphate transport is translated into changes in arbuscule fate.

#### 4.2.3 Transcription factor network regulating arbuscule senescence

In addition to the transport activity of PT4 and PT11, specific transcription factors are directly involved in the regulatory program of arbuscule senescence. In contrast to positive regulators such as RAM1 and WRI5a, which promote arbuscule maturation, certain transcription factors appear to function as “switches” for senescence. In *Medicago truncatula*, the R2R3-MYB transcription factor MtMYB1 has been identified as a potential promoter of arbuscule senescence. MtMYB1 is specifically upregulated in cortical cells containing senescing arbuscules; its overexpression leads to premature arbuscule degeneration, whereas its knockout delays the senescence process, suggesting it acts as an executor within the degeneration program. MtMYB1 is associated with the transcriptional program of arbuscule degeneration, whereas the upstream signals connecting phosphate status, PT4 activity, and MtMYB1 remain incompletely understood. This counterintuitive regulatory pattern characterized by maintenance under low phosphate and initiation under high phosphate (contrary to the typical paradigm where phosphate starvation promotes uptake and phosphate sufficiency promotes senescence), further highlights the complexity of signal interpretation within the interfacial microenvironment (Floss et al., 2017**;** Paries et al., 2025**;** Jiang et al., 2018).

After arbuscules fully degenerate, the cortical cells do not die but instead revert to a non-symbiotic state; they may even be re-colonized by new fungal hyphae forming new arbuscules within the same cell a process known as recolonization of previously colonized cortical cells. This phenomenon suggests that the termination of arbuscule senescence is an active, reversible cellular cleanup process rather than irreversible cell death. Plants degrade the degenerated fungal remnants via the lysosomal pathway and recycle membrane components, thereby preparing for potential re-infection. Following senescence, the expression levels of PT4 and PT11 drop to basal levels, and their reactivation depends on renewed fungal contact and the re-perception of signals (Kokkoris, 2026**;** Russo and Genre, 2021).

In summary, the precise regulation of arbuscule aging holds significant adaptive importance. By actively limiting the lifespan of individual arbuscules, plants can flexibly adjust their investment in symbiotic relationships based on environmental changes (when phosphate supply is sufficient), thereby avoiding excessive allocation to symbiotic units with diminishing marginal benefits. Additionally, the periodic replacement of arbuscules provides plants with opportunities to reassess the value of current symbiotic partners and engage in renegotiation. This cyclical strategy of evaluating, maintaining, or terminating individual arbuscules may allow plants to dynamically optimize AM symbiosis throughout development.

### 4.3 Bidirectional communication at the arbuscular interface

In the preceding two sections, we examined how *PT4* and *PT11* loss of function accelerates arbuscule degeneration. These discussions raise a fundamental question: what determines the fate of an arbuscule, its maintenance or its decline? Growing evidence suggests that the answer lies in the continuous bidirectional signaling dialogue occurring at the peri-arbuscular interface. The essence of the AM symbiosis is the bidirectional exchange of carbon and phosphorus; the continuity and fairness of this exchange rely on precise signaling and reciprocal monitoring between the plant and the fungus at the interface (Kiers et al., 2011**;** Zhang et al., 2023). Therefore, this chapter details how the bidirectional exchange of carbon and phosphorus regulates arbuscule maintenance.

#### 4.3.1 Molecular communication at the arbuscular interface

The arbuscular interface—specifically the PAS, situated between the PAM and the fungal plasma membrane, serves not only as a site for nutrient exchange but also as a dynamic interface for molecular communication between the plant and fungal partners. A continuous molecular dialogue persists between the plant and the fungus even during the mature stage of symbiosis. Fungal-derived signaling molecules known as Myc factors continue to function during the arbuscule maturation phase. For instance, the LCO receptor LjLYS11 in *Lotus japonicus* is specifically expressed in cortical cells containing arbuscules, suggesting that fungal LCO signals are still perceived by the plant during this stage. On the plant side, recent genetic and biochemical studies have identified MtLYK8 as an important component of chitin oligosaccharide perception during AM symbiosis. MtLYK8 is functionally redundant with MtCERK1 for AM fungal colonization but appears to have a more specific role in symbiotic signaling, as its disruption does not substantially affect chitin-triggered immune responses. MtLYK8 interacts with MtCERK1 and DMI2, and these interactions are enhanced by chitin oligosaccharides, supporting a model in which an MtLYK8–MtCERK1–DMI2 receptor complex contributes to the selective activation of symbiosis signaling (Zhang et al., 2024**;** Rodriguez-Garcia and Müller, 2026**;** Rasmussen et al., 2016). This finding provides an important example of how plants may distinguish mutualistic fungal signals from immune-inducing signals at the root–fungus interface.

#### 4.3.2 PT4/PT11-mediated phosphate transport as a potential determinant of interfacial physiological conditions

PT4 and PT11 occupy a strategically important position at the PAM, where they mediate the uptake of phosphate released into the symbiotic interface. Their activity is therefore expected to influence phosphate flux across the plant–fungus interface and may contribute to shaping local physiological conditions within the PAS. When PT4/PT11 function efficiently, rapid phosphate uptake by the host could help maintain a proposed low-phosphate interfacial environment on the plant side of the exchange interface and thereby sustain a potentially favorable phosphate gradient for continued phosphate transfer from the fungal partner. Conversely, impaired transporter activity is expected to alter phosphate flux and may disrupt the nutritional balance associated with reciprocal exchange. Importantly, these potential effects should not be interpreted as evidence that PT4/PT11 themselves act as canonical signaling proteins. At present, there is no direct evidence demonstrating that PT4/PT11 transmit intracellular signals through transport-independent conformational signaling, interact directly with downstream signaling proteins, or function as receptors or dedicated signaling hubs. Likewise, whether changes in interfacial phosphate concentration are directly sensed by either the plant or the fungus to control arbuscule fate remains unresolved (Javot et al., 2007a**;** Yang et al., 2012**;** Ikeh et al., 2016**;** Bhalla et al., 2022**;** Harrison et al., 2002**;** Zhang et al., 2023).

We therefore propose that PT4/PT11-mediated phosphate transport may influence arbuscule maintenance indirectly through its effects on nutrient flux and the physiological state of the symbiotic interface. Altered transporter activity could potentially affect local phosphate availability, fungal phosphate release, host phosphate status, carbon–phosphate exchange balance, or other metabolic processes associated with symbiotic performance. Distinguishing among these possibilities will require direct measurements of phosphate dynamics at the periarbuscular interface and experiments that separate transport activity from potential non-transport functions. Accordingly, the proposed link between PT4/PT11-mediated phosphate transport and arbuscule maintenance should be distinguished from direct receptor-like or transport-independent signaling by the transporters themselves. Importantly, fungal phosphate release itself may represent an additional regulatory step in determining interfacial phosphate availability. The identification of the SPX-domain-containing RiPT7 and the proposed involvement of fungal PHO1/SYG1-type proteins indicate that phosphate delivery to the PAS is likely controlled by active fungal transport processes rather than being a passive consequence of polyphosphate hydrolysis (Ezawa and Saito, 2018**;** Xie et al., 2022**;** Duan et al., 2024).

#### 4.3.3 The arbuscular interface signal sensing and response mechanism

Plants possess complex sensing networks at the interface. Cell wall components, receptor kinases, signaling peptides, and extracellular vesicles collectively constitute this sensing system. Notably, the cell wall integrity sensing pathway has been shown to regulate arbuscule maintenance (Rodriguez-Garcia and Müller, 2026).

Furthermore, the feedback regulation of nutritional signaling is tightly coupled with bidirectional signaling at the interface. Phosphate-starvation-responsive PHR transcription factors not only directly activate *PT4*/*PT11* transcription via P1BS elements but also regulate SL biosynthesis genes, thereby influencing signal output during the early stages of symbiosis establishment. Meanwhile, SPX proteins act as important components of phosphate-status sensing; under phosphate-sufficient conditions, InsP8-dependent SPX–PHR interactions suppress phosphate-starvation-responsive transcription, including the expression of symbiosis-associated phosphate transporter genes such as *PT4*/*PT11*. This circuit establishes a global regulatory network that integrates the plant’s overall nutritional status with local signaling at the interface (Das et al., 2022**;** Das and Gutjahr, 2022**;** Shi et al., 2021**;** Liao et al., 2022). An imbalance in bidirectional nutrient exchange may contribute to arbuscule degeneration. When PT4/PT11 function is impaired, reduced phosphate transfer may alter the nutritional balance between the plant and fungus and could be accompanied by changes in carbon allocation, fungal metabolic activity, or host defense responses. These changes may subsequently influence senescence-associated pathways, including the induction of transcription factors such as MtMYB1. However, whether such responses reflect a direct plant sensing mechanism, a consequence of altered fungal metabolism, or secondary effects of impaired membrane and nutrient exchange remains unresolved (Floss et al., 2017**;** Paries et al., 2025**;** Jiang et al., 2018**;** Bashyal et al., 2025).

Collectively, at least four non-mutually-exclusive mechanisms could account for the accelerated arbuscule senescence observed in *pt4* and *pt11* mutants: (1) interfacial phosphate accumulation disrupting the local physicochemical environment and signaling; (2) fungal carbon starvation resulting from uncoupled carbon-phosphate exchange; (3) loss of PAM integrity leading to structural collapse; and (4) localized activation of plant immune responses. The relative contributions of these mechanisms likely vary depending on the developmental stage, plant species, and environmental conditions. Critically, these possibilities remain to be systematically tested, and no single mechanism has been conclusively demonstrated as the primary cause of the *pt4* mutant phenotype (**Table 3**).

**Table 3:** Working models linking PT4/PT11-mediated phosphate transport to arbuscule senescence.

Hypothesis	Key supporting evidence	Outstanding questions / Current limitations	Testable predictions	Reference
Interfacial phosphate accumulation	Loss of *PT4*/*PT11* reduces phosphate uptake and is associated with premature arbuscule degeneration; altered phosphate flux could modify the interfacial environment.	Direct measurements of phosphate concentration in the PAS of PT4/PT11-deficient cells are lacking; direct sensing of interfacial phosphate has not been demonstrated.	Quantitative imaging or biosensors should detect altered PAS phosphate dynamics in PT4/PT11-deficient arbuscules.	(Javot et al., 2007a; Javot et al., 2007b; Yang et al., 2012; Chiu and Paszkowski, 2019)
Fungal carbon starvation	AM symbiosis depends on reciprocal carbon–phosphate exchange; impaired phosphate return could reduce the nutritional benefit to the plant and alter carbon allocation to the fungus.	Direct evidence linking *PT4*/*PT11* loss to fungal carbon starvation during arbuscule senescence remains limited.	PT4/PT11 deficiency should be associated with altered fungal carbon status and reduced carbon transfer before arbuscule degeneration.	(Javot et al., 2011; Jiang et al., 2018; MacLean et al., 2017; Breuillin-Sessoms et al., 2015)
PAM/membrane integrity defects	PT4/PT11-deficient roots display abnormalities at the plant–fungus interface, and PAM integrity is essential for functional nutrient exchange.	It remains unclear whether membrane defects are a primary consequence of impaired phosphate transport or a secondary feature of arbuscule degeneration.	Alterations in PAM integrity should precede or quantitatively correlate with arbuscule collapse.	(Zhang et al., 2015; Ivanov and Harrison, 2014)
Localized immune activation	AM symbiosis requires suppression or modulation of host immune responses, and perturbation of the arbuscular interface can activate defense-related pathways.	Direct evidence that *PT4*/*PT11* loss specifically triggers immune activation that causes arbuscule senescence is lacking.	PT4/PT11 deficiency should increase defense-related transcription or immune signaling before arbuscule degeneration.	(Floss et al., 2017; Rodriguez-Garcia and Müller, 2026)

## 5 MtPT4 and OsPT11: a comparison of symbiosis-specific phosphate transporters and their regulatory mechanisms

Similar to MtPT4, which functions as a symbiosis-specific phosphate transporter for AM symbiosis, OsPT11 in rice is a P_i_ transporter that is a symbiosis-specific phosphate transporter during AM symbiosis. OsPT11 is localized to root cells containing arbuscules and is responsible for mediating the transport of phosphate from the fungus to the plant. Functional studies have shown that *Ospt11* mutants completely lose their ability to absorb P_i_ mediated by AMF; isotope tracing indicates that OsPT11 contributes to more than 70% of P_i_ uptake in mycorrhizal rice (Du et al., 2026).

At the transcriptional regulation level, rice possesses an AM symbiosis transcriptional regulatory network centered on OsPHR2. Yeast one-hybrid screening revealed that OsPHR2 directly binds to and activates the promoters of several key AM symbiosis genes, including the transcription factors OsRAM1 and OsWRI5A, as well as the phosphate transporter OsPT11 and the ammonium transporter OsAMT3;1. OsPHR1/2/3 play critical and redundant roles in AM symbiosis, the mycorrhizal colonization level in *Osphr2* single mutants is significantly reduced, while the *Osphr1/2/3* triple mutants almost completely block AM fungal colonization (Shi et al., 2021).

Regarding the RAM1-WRI5a regulatory module, rice and *Medicago truncatula* exhibit both conservation and differences. Conservation is reflected in the following: (1) The absence of both *OsRAM1* and *OsWRI5a/b* leads to a significant reduction in mycorrhizal symbiosis in rice; (2) RAM1 directly activates its target gene *RAM2* (encoding glycerol-3-phosphate acyltransferase); RAM2 is essential for arbuscule formation and lipid export, and its mutation leads to abnormal arbuscule development; (3) WRI5a and RAM1 synergistically regulate downstream genes required for arbuscule development. However, the upstream regulation of this module in rice exhibits a more complex hierarchical structure. OsIDD7, a newly identified central transcriptional regulator, acts in concert with SLR1 and PHR2 to simultaneously activate the promoters of multiple key AM symbiosis genes, including *PT11*, *RAM1*, *WRI5a*, and *CYCLOPS*. Furthermore, the promoters of both *OsRAM1* and *OsWRI5a* contain at least two P1BS elements, which are direct targets of OsPHR2, forming a regulatory hierarchy of PHR2 → RAM1/WRI5a → downstream effector genes. No similar function has been reported for IDD7 homologs in *Medicago truncatula*, suggesting that this regulatory hierarchy may be specific to rice (Shi et al., 2021**;** Chen et al., 2026).

Significant differences also exist between rice and *Medicago truncatula* in terms of hormonal regulation. SLR1 a key repressor of GA signaling, is directly involved in the transcriptional activation of *OsPT11*: IDD7 physically interacts with SLR1 and PHR2 via its conserved TQDFLG domain, and the three proteins synergistically activate the *OsPT11* promoter. This forms an interesting parallel to the mechanism in *Medicago truncatula*, where DELLA forms a complex with CCaMK-CYCLOPS to activate *MtPT4*—both involve DELLA proteins acting as coactivators, but in rice, SLR1 acts in synergy with IDD7 and PHR2 rather than being directly coupled to the CCaMK-CYCLOPS pathway. At the level of P_i_ signal feedback regulation, SPX proteins act as repressors of PHRs to negatively regulate AM symbiosis: the *Osspx1/2/3/5* quadruple mutant attenuates the inhibitory effect of high P_i_ on AM symbiosis, indicating that high P_i_ inhibits AM symbiosis in part through the PHR-SPX module (Shi et al., 2021**;** Chen et al., 2026**;** Wang et al., 2009).

In summary, OsPT11 is functionally highly similar to MtPT4 in *Medicago truncatula* but exhibits rice-specific complexity in the structure of its transcriptional regulatory network. It is activated, and the region upstream of the RAM1-WRI5a module is directly regulated by OsPHR2, forming a more multi-layered regulatory hierarchy.

## 6 Future perspectives

This review summarizes current evidence indicating that PT4 and PT11 are key components of the mycorrhizal phosphate uptake pathway and that their transport activity is closely associated with nutrient exchange and arbuscule maintenance. Their expression is regulated by interconnected symbiotic, nutritional, hormonal, and developmental pathways, whereas loss of transporter function is associated with impaired phosphate acquisition and premature arbuscule degeneration. These observations provide a strong basis for considering PT4/PT11 as important functional nodes at the plant–fungus nutrient exchange interface. However, the available evidence does not establish PT4/PT11 as independent signaling proteins or direct regulators of arbuscule developmental programs. Several important questions therefore remain unresolved, particularly regarding how transporter activity, interfacial nutrient status, and arbuscule fate are mechanistically connected. Addressing these questions will require approaches that can distinguish transport-dependent effects from potential transport-independent functions.

### 6.1 Testing potential transport-independent functions and the PT4/PT11 interactome

Current research primarily attributes the role of PT4/PT11 in maintaining arbuscule maturity to their phosphate transport activity. As integral membrane proteins located on the PAM, do the intracellular domains of PT4/PT11 interact directly with signaling proteins? How is their transport activity precisely regulated to adapt to real-time fluctuations in phosphate concentration at the interface? What mechanisms govern their polar localization on the PAM? Future research combining proximity labeling techniques (such as TurboID) with mass spectrometry holds the promise of systematically characterizing the PT4/PT11 interactome, identifying potential interacting proteins and determining whether any such interactions have functional consequences beyond phosphate transport.

### 6.2 Key transporter component and cellular decision-making mechanisms in arbuscule senescence

While it is known that the loss of PT4/PT11 accelerates senescence, a process involving transcription factors such as MtMYB1, several questions remain unresolved: (1) Is arbuscule senescence a program actively initiated by the plant, or is it the result of the fungus actively degenerating due to a lack of carbon sources? (2) What is the cell-autonomous molecular basis for the phenomenon where adjacent arbuscules within the same root segment exist at different developmental stages? (3) Recent advances in single-cell and spatial transcriptomic technologies have already begun to resolve the cellular and developmental heterogeneity of AM symbiosis. Spatial co-transcriptomic analysis of *Medicago truncatula* roots has identified discrete transcriptional states associated with different stages of arbuscule development and enabled simultaneous characterization of host and fungal transcriptional programs at the symbiotic interface (Serrano et al., 2024). These approaches move beyond bulk-root transcriptomics by resolving cell-type- and stage-specific regulatory programs and therefore provide an important framework for investigating how *PT4*/*PT11* expression, phosphate transport, membrane trafficking, and arbuscule maintenance are coordinated at cellular resolution. Future integration of spatial transcriptomics, single-cell sequencing, live-cell imaging, and quantitative nutrient flux measurements may further reveal how transcriptional states are translated into dynamic changes in the arbuscular interface.

### 6.3 Systematic identification and functional validation of inter-kingdom signaling molecules

Beyond LCOs and COs, do fungi release other signaling molecules such as effector proteins, small RNAs, or metabolites that influence PT4/PT11 expression or arbuscule lifespan? Do plants transmit regulatory signals to fungi via extracellular vesicles? Given the recent discovery in root nodule symbiosis that plant-derived miRNAs can enter rhizobial cells via extracellular vesicles to regulate gene expression, investigating whether a similar mechanism exists in AM symbiosis is highly valuable. Integrating multi-omics analyses such as secretomics, metabolomics, and extracellular vesicle profiling with fluorescence-based cross-kingdom tracking technologies promises to systematically reveal this largely unexplored inter-kingdom signaling network.

### 6.4 From module analysis to system modeling: integrating multi-level regulatory frameworks

Future research should establish multi-scale dynamic models that incorporate signal perception, transcriptional regulation, metabolic flux (carbon/phosphate exchange), and developmental dynamics into a unified computational framework. Such models would simulate trends in arbuscule lifespan and phosphorus uptake efficiency under varying environmental conditions (e.g., phosphate levels, light intensity, and hormonal treatments). This systems biology approach will propel the field from a descriptive phase focused on identifying individual genes toward an engineering-oriented phase focused on predicting and designing symbiotic performance.

### 6.5 Translational applications for sustainable agriculture

The translational potential of AM symbiosis is increasingly supported by field-based evidence, although its agronomic benefits remain strongly dependent on crop genotype, fungal identity, soil fertility, and management practices. Recent field experiments have demonstrated that AM fungal inoculation can improve phosphorus acquisition and crop performance under reduced fertilizer inputs. For example, field inoculation of potato at the seedling stage increased AM colonization and phosphorus uptake and maintained yield under a 25% reduction in phosphorus fertilizer, while increasing phosphorus partial factor productivity by approximately 39% (Liu et al., 2025). Similarly, field experiments in rice demonstrated that AMF inoculation could partially mitigate yield losses associated with alternate wetting and drying irrigation and reduced NPK fertilization, although the magnitude of the response depended on irrigation regime, fertilizer level, and rice genotype (Mbodj et al., 2025). Long-term field experiments further indicate that N and P fertilization thresholds strongly influence AM fungal communities and crop productivity, emphasizing the importance of optimizing fertilizer inputs together with mycorrhizal activity rather than considering AM fungi independently of soil nutrient management (Liu et al., 2024b).

These findings support the potential of AM symbiosis as a component of sustainable phosphorus management, but they also highlight substantial context dependence. From the perspective of PT4/PT11, a major translational challenge is therefore to determine whether genetic or agronomic strategies that enhance mycorrhiza-specific phosphate transport can provide consistent benefits across diverse field environments. Future strategies should combine AM fungal inoculation, optimized phosphorus fertilization, crop genotype selection, and manipulation of symbiosis-associated regulatory pathways. Such approaches may ultimately improve phosphorus-use efficiency while minimizing unnecessary phosphorus fertilizer inputs. However, direct field-level evidence linking PT4/PT11 activity to agronomic performance remains limited, and this gap should be explicitly addressed before transporter-based strategies can be translated into crop improvement programs (**Figure 3**).

**Figure 3:**
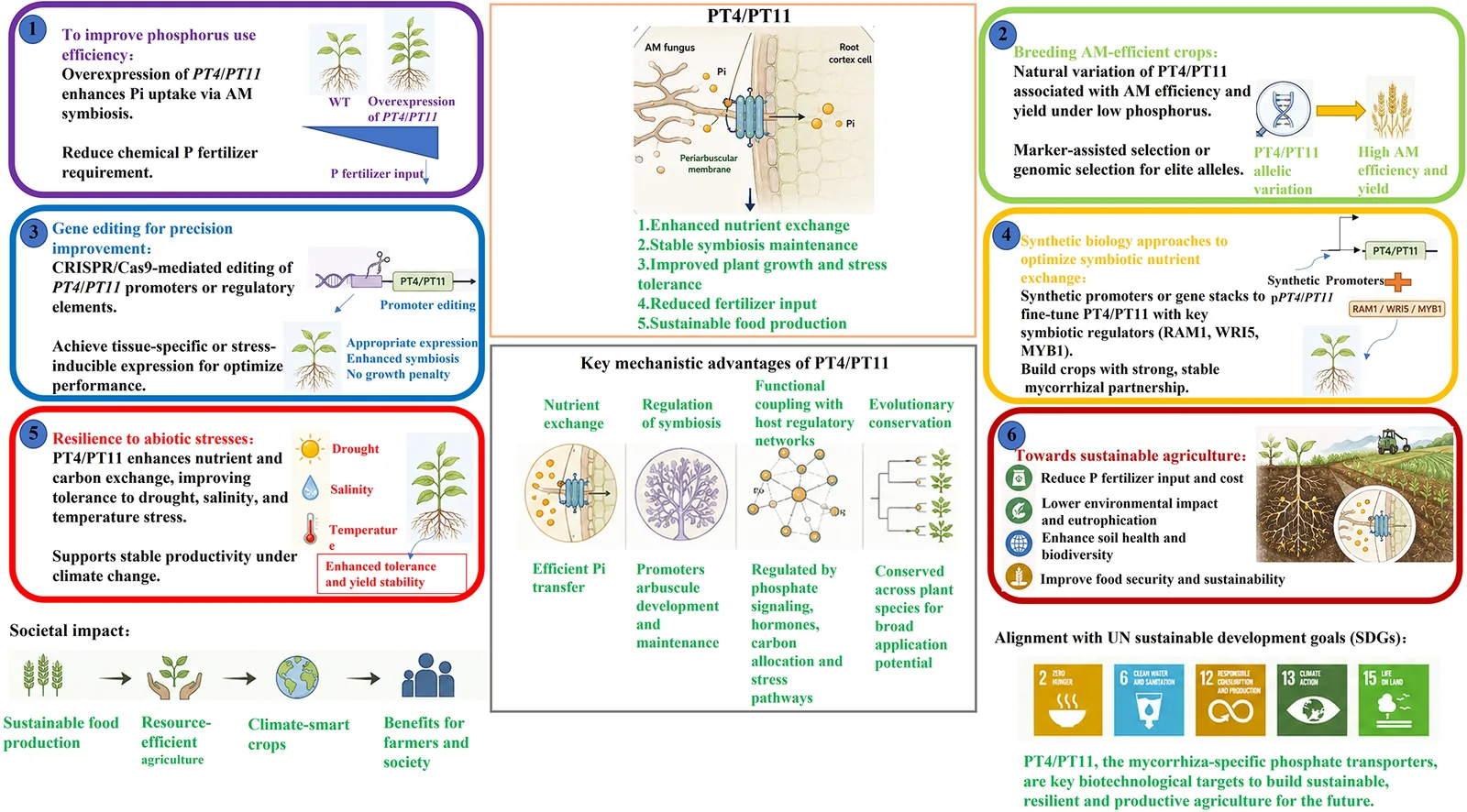
Transformative applications of mycorrhiza-specific phosphate transporters PT4/PT11 in sustainable agriculture. PT4/PT11-mediated phosphate uptake at the arbuscular interface provides a potential molecular target for improving the efficiency and stability of arbuscular mycorrhizal (AM) symbiosis. Six complementary application strategies are highlighted. (1) Improving phosphorus-use efficiency: enhancing *PT4*/*PT11* expression or promoter activity may increase mycorrhiza-mediated phosphate acquisition and potentially reduce dependence on mineral phosphate fertilizers. (2) Breeding AM-efficient crops: natural allelic variation in PT4/PT11 and associated regulatory components could be exploited through marker-assisted selection, genomic selection, or other breeding approaches to develop crops with improved AM responsiveness and phosphorus-use efficiency. (3) Precision genome editing: CRISPR/Cas-based editing of *PT4*/*PT11* promoters or regulatory elements may enable tissue-specific, developmentally regulated, or stress-responsive expression while minimizing potential growth penalties. (4) Synthetic biology approaches to optimize symbiotic nutrient exchange: synthetic promoters, gene stacking, or targeted manipulation of PT4/PT11 together with key symbiotic regulators, such as RAM1, WRI5, and MYB-family transcription factors, may provide opportunities to optimize the strength and stability of AM associations. (5) Enhancing abiotic-stress resilience: improved phosphate and carbon exchange mediated by functional PT4/PT11 pathways may contribute to plant tolerance to drought, salinity, and temperature stress and support yield stability under changing environmental conditions. (6) Sustainable agriculture: integration of PT4/PT11 strategies with AM inoculation, precision breeding, genome editing, and improved nutrient management could contribute to reduced phosphate fertilizer inputs, lower environmental impacts, improved soil health and biodiversity, and more resource-efficient crop production. Together, these strategies illustrate the potential of PT4/PT11 as molecular targets for translating mechanistic insights into AM symbiosis into practical strategies for sustainable and climate-resilient agriculture.

## 7 Conclusion

PT4 and PT11 are key functional components of the mycorrhizal phosphate uptake pathway and occupy a strategically important position at the plant–fungus nutrient exchange interface. Available genetic and physiological evidence demonstrates that their activity is required for efficient symbiotic phosphate acquisition and is closely linked to the maintenance of functional arbuscules. These findings support a strong functional coupling between phosphate transport, nutrient exchange, and symbiotic stability. However, the current evidence does not establish PT4/PT11 as canonical signaling proteins or demonstrate a transport-independent mechanism through which they directly interact with downstream signaling components. Accordingly, the influence of PT4/PT11 on arbuscule maintenance is most conservatively interpreted as arising primarily from their transport-dependent effects on phosphate flux and the physiological conditions of the symbiotic interface.

A major challenge for future research will be to determine how altered PT4/PT11 activity is translated into changes in arbuscule fate. Possible mechanisms include changes in local phosphate availability, host nutrient status, carbon–phosphate exchange balance, fungal metabolic activity, or additional plant–fungus sensing systems. These mechanisms should currently be regarded as alternative or complementary working models rather than established signaling pathways. Combining transport-defective separation-of-function mutants, quantitative measurements of interfacial phosphate dynamics, cell-specific omics, proximity labeling, and targeted protein-interaction analyses will be essential to distinguish transport-dependent effects from possible transport-independent functions. Such experiments will provide a more rigorous mechanistic framework for understanding how symbiotic phosphate transport contributes to the maintenance and regulation of arbuscular mycorrhizal symbiosis.

Recent advances in spatial and single-cell transcriptomics, live-cell imaging, and field-based agronomic studies are beginning to connect molecular regulation with cellular and ecological outcomes of AM symbiosis. These approaches provide new opportunities to determine how PT4/PT11-mediated phosphate transport is coordinated with receptor-mediated signal perception, membrane trafficking, nutrient exchange, and arbuscule maintenance across developmental stages and environmental conditions. Ultimately, integrating mechanistic studies with field validation will be essential for translating knowledge of PT4/PT11 and AM symbiosis into strategies for improving phosphorus-use efficiency and sustainable crop production.

## References

[B1] Agisha V. N. Suraby E. J. Dhandapani S. Sng Y. H. Lim S. H. Park B. S. (2024). Molecular mechanisms of phosphate use efficiency in Arabidopsis via Penicillium olsonii TLL1. Int. J. Mol. Sci. 25, 12865. doi: 10.3390/ijms252312865 39684576 PMC11640997

[B2] Ahmed N. Li J. Li Y. Deng L. Deng L. Chachar M. . (2025). Symbiotic synergy: How arbuscular mycorrhizal fungi enhance nutrient uptake, stress tolerance, and soil health through molecular mechanisms and hormonal regulation. IMA Fungus 16, e144989. doi: 10.3897/imafungus.16.144989 40162002 PMC11953731

[B3] Aizaz M. Lubna Jan R. Asaf S. Bilal S. Kim K. M. . (2024). Regulatory dynamics of plant hormones and transcription factors under salt stress. Biol. (Basel) 13, 673. doi: 10.3390/biology13090673 39336100 PMC11429359

[B4] Akiyama K. Matsuzaki K. Hayashi H. (2005). Plant sesquiterpenes induce hyphal branching in arbuscular mycorrhizal fungi. Nature 435, 824–827. doi: 10.1038/nature03608 15944706

[B5] Alexander T. Toth R. Meier R. Weber H. C. (1989). Dynamics of arbuscule development and degeneration in onion, bean, and tomato with reference to vesicular–arbuscular mycorrhizae in grasses. Can. J. Bot. 67, 2505–2513. doi: 10.1139/b89-32034819996

[B6] Ansari R. A. Egamberdievich K. E. Fayziyevich B. X. Kurbonovich T. M. Enverovna B. L. Raximovna M. T. . (2026). Soil to cytoplasm: The mycorrhizal phosphorus express and its regulatory steps. J. Fungi 12, 473. doi: 10.3390/jof12070473 42506235 PMC13412362

[B7] Antolín-Llovera M. Petutschnig E. Oldroyd G. E. D. (2014). Knowing your friends and foes—plant receptor-like kinases as initiators of symbiosis or defence. New Phytol. 204, 791–802. doi: 10.1111/nph.13117 25367611

[B8] Balzergue C. Chabaud M. Barker D. G. Bécard G. Rochange S. F. (2013). High phosphate reduces host ability to develop arbuscular mycorrhizal symbiosis without affecting root calcium spiking responses to the fungus. Front. Plant Sci. 4, 426. doi: 10.3389/fpls.2013.00426 24194742 PMC3810610

[B9] Balzergue C. Puech-Pagès V. Bécard G. Rochange S. F. (2010). The regulation of arbuscular mycorrhizal symbiosis by phosphate in pea involves early and systemic signalling events. J. Exp. Bot. 62, 1049–1060. doi: 10.1093/jxb/erq335 21045005 PMC3022399

[B10] Bari R. Datt Pant B. Stitt M. Scheible W. R. (2006). PHO2, microRNA399, and PHR1 define a phosphate-signaling pathway in plants. Plant Physiol. 141, 988–999. doi: 10.1104/pp.106.079707 16679424 PMC1489890

[B11] Bashyal S. Everett H. Matsuura S. Müller L. M. (2025). A plant CLE peptide and its fungal mimic promote arbuscular mycorrhizal symbiosis via CRN-mediated ROS suppression. Proc. Natl. Acad. Sci. U.S.A. 122, e2422215122. doi: 10.1073/pnas.2422215122 40228122 PMC12037060

[B12] Bayle V. Arrighi J. F. Creff A. Nespoulous C. Vialaret J. Rossignol M. . (2011). Arabidopsis thaliana high-affinity phosphate transporters exhibit multiple levels of posttranslational regulation. Plant Cell 23, 1523–1535. doi: 10.1105/tpc.110.081067 21521698 PMC3101552

[B13] Benedetto A. Magurno F. Bonfante P. Lanfranco L. (2005). Expression profiles of a phosphate transporter gene (GmosPT) from the endomycorrhizal fungus Glomus mosseae. Mycorrhiza 15, 620–627. doi: 10.1007/s00572-005-0006-9 16133249

[B14] Bhalla K. Qu X. Kretschmer M. Kronstad J. W. (2022). The phosphate language of fungi. Trends Microbiol. 30, 338–349. doi: 10.1016/j.tim.2021.08.002 34479774 PMC8882699

[B15] Breuillin-Sessoms F. Floss D. S. Gomez S. K. Pumplin N. Ding Y. Levesque-Tremblay V. . (2015). Suppression of arbuscule degeneration in Medicago truncatula phosphate transporter4 mutants is dependent on the ammonium transporter 2 family protein AMT2;3. Plant Cell 27, 1352–1366. doi: 10.1105/tpc.114.131144 25841038 PMC4558683

[B16] Brundrett M. (2004). Diversity and classification of mycorrhizal associations. Biol. Rev. Camb Philos. Soc 79, 473–495. doi: 10.1017/s1464793103006316 15366760

[B17] Bücking H. Heyser W. (2001). Microautoradiographic localization of phosphate and carbohydrates in mycorrhizal roots of Populus tremula× Populus alba and the implications for transfer processes in ectomycorrhizal associations. Tree Physiol. 21, 101–107. doi: 10.1093/treephys/21.2-3.101 11303640

[B18] Carrión C. A. Martínez D. Costa M. L. Guiamet J. J. (2014). Senescence-associated vacuoles, a specific lytic compartment for degradation of chloroplast proteins? Plants (Basel) 3, 498–512. doi: 10.3390/plants3040498 PMC4844279 27135516

[B19] Chakravarthy S. Tuori R. P. D'Ascenzo M. D. Fobert P. R. Despres C. Martin G. B. (2003). The tomato transcription factor Pti4 regulates defense-related gene expression via GCC box and non-GCC box cis elements. Plant Cell 15, 3033–3050. doi: 10.1105/tpc.017574 14630974 PMC282854

[B20] Charpentier M. Sun J. Martins T. V. Radhakrishnan G. V. Findlay K. Soumpourou E. . (2016). Nuclear-localized cyclic nucleotide-gated channels mediate symbiotic calcium oscillations. Science 352, 1102–1105. doi: 10.1126/science.aae0109 27230377

[B21] Chen A. Gu M. Sun S. Zhu L. Hong S. Xu G. (2011). Identification of two conserved cis-acting elements, MYCS and P1BS, involved in the regulation of mycorrhiza-activated phosphate transporters in eudicot species. New Phytol. 189, 1157–1169. doi: 10.1111/j.1469-8137.2010.03556.x 21106037

[B22] Chen A. Gu M. Wang S. Chen J. Xu G. (2018). Transport properties and regulatory roles of nitrogen in arbuscular mycorrhizal symbiosis. Semin. Cell Dev. Biol. 74, 80–88. doi: 10.1016/j.semcdb.2017.06.015 28647533

[B23] Chen A. Hu J. Sun S. Xu G. (2007). Conservation and divergence of both phosphate- and mycorrhiza-regulated physiological responses and expression patterns of phosphate transporters in solanaceous species. New Phytol. 173, 817–831. doi: 10.1111/j.1469-8137.2006.01962.x 17286830

[B24] Chen P. J. Tu Y. T. Chang Y. T. Ho M. C. Kuo W. N. Guo Z. L. . (2026). OsIDD7 integrates signaling networks for arbuscular mycorrhizal symbiosis. Nat. Commun. 17, 77162. doi: 10.1038/s41467-026-77162-3 42816479 PMC13627655

[B25] Chen Q. Song Y. An Y. Lu Y. Zhong G. (2024a). Soil microorganisms: Their role in enhancing crop nutrition and health. Diversity 16, 734. doi: 10.3390/d1612073430654563

[B26] Chen X. Bai Y. Lin Y. Liu H. Han F. Chang H. . (2024b). Genome-wide identification and characterization of the PHT1 gene family and its response to mycorrhizal symbiosis in Salvia miltiorrhiza under phosphate stress. Genes (Basel) 15, 589. doi: 10.3390/genes15050589 38790218 PMC11120713

[B27] Chen X. Chen J. Liao D. Ye H. Li C. Luo Z. . (2022). Auxin-mediated regulation of arbuscular mycorrhizal symbiosis: A role of SlGH3.4 in tomato. Plant Cell Environ. 45, 955–968. doi: 10.1111/pce.14210 34713922

[B28] Chiu C. H. Paszkowski U. (2019). Mechanisms and impact of symbiotic phosphate acquisition. Cold Spring Harb. Perspect. Biol. 11, a034603. doi: 10.1101/cshperspect.a034603 30910773 PMC6546048

[B29] Choi J. Summers W. Paszkowski U. (2018). Mechanisms underlying establishment of arbuscular mycorrhizal symbioses. Annu. Rev. Phytopathol. 56, 135–160. doi: 10.1146/annurev-phyto-080516-035521 29856935

[B30] Cox G. Tinker P. B. (1976). Translocation and transfer of nutrients in vesicular–arbuscular mycorrhizas: I. The arbuscule and phosphorus transfer: a quantitative ultrastructural study. New Phytol. 77, 371–378. doi: 10.1111/j.1469-8137.1976.tb01526.x

[B31] Das D. Gutjahr C. (2022). Old dog, new trick: The PHR-SPX system regulates arbuscular mycorrhizal symbiosis. Mol. Plant 15, 225–227. doi: 10.1016/j.molp.2021.12.010 34968731

[B32] Das D. Paries M. Hobecker K. Gigl M. Dawid C. Lam H. M. . (2022). Phosphate starvation response transcription factors enable arbuscular mycorrhiza symbiosis. Nat. Commun. 13, 477. doi: 10.1038/s41467-022-27976-8 35078978 PMC8789775

[B33] Delaux P. M. Gutjahr C. (2024). Evolution of small molecule-mediated regulation of arbuscular mycorrhiza symbiosis. Philos. Trans. R. Soc Lond. B. Biol. Sci. 379, 20230369. doi: 10.1098/rstb.2023.0369 39343030 PMC11439497

[B34] Díaz V. Villalobos M. Arriaza K. Flores K. Hernández-Saravia L. P. Velásquez A. (2025). Decoding the dialog between plants and arbuscular mycorrhizal fungi: A molecular genetic perspective. Genes (Basel) 16, 143. doi: 10.3390/genes16020143 PMC11855461 40004472

[B35] Dindas J. DeFalco T. A. Yu G. Zhang L. David P. Bjornson M. . (2022). Direct inhibition of phosphate transport by immune signaling in Arabidopsis. Curr. Biol. 32, 488–495. doi: 10.1016/j.cub.2021.11.063 34919806 PMC8791604

[B36] Dong J. Ma G. Sui L. Wei M. Zhang Y. Li S. . (2019). Inositol pyrophosphate InsP8 acts as an intracellular phosphate signal in plants. Mol. Plant 12, 1463–1473. doi: 10.1016/j.molp.2019.08.002 31419530

[B37] Dreyer I. Spitz O. Kanonenberg K. Montag K. Handrich M. R. Ahmad S. . (2019). Nutrient exchange in arbuscular mycorrhizal symbiosis from a thermodynamic point of view. New Phytol. 222, 1043–1053. doi: 10.1111/nph.15646 30565261 PMC6667911

[B38] Du Z. Guan Z. Liu H. Zhang J. He H. Zheng Z. . (2026). Cryo-EM structure and dynamic basis of phosphate uptake by PHT1 in rice. Dev. Cell 61, 164–177. doi: 10.1016/j.devcel.2025.09.003 41005295

[B39] Duan S. Feng G. Limpens E. Bonfante P. Xie X. Zhang L. (2024). Cross-kingdom nutrient exchange in the plant–arbuscular mycorrhizal fungus–bacterium continuum. Nat. Rev. Microbiol. 22, 773–790. doi: 10.1038/s41579-024-01073-7 39014094

[B40] Etemadi M. Gutjahr C. Couzigou J. M. Zouine M. Lauressergues D. Timmers A. . (2014). Auxin perception is required for arbuscule development in arbuscular mycorrhizal symbiosis. Plant Physiol. 166, 281–292. doi: 10.1104/pp.114.246595 25096975 PMC4149713

[B41] Ezawa T. Saito K. (2018). How do arbuscular mycorrhizal fungi handle phosphate? New insight into fine-tuning of phosphate metabolism. New Phytol. 220, 1116–1121. doi: 10.1111/nph.15187 29701874

[B42] Fitze D. Wiepning A. Kaldorf M. Ludwig-Müller J. (2005). Auxins in the development of an arbuscular mycorrhizal symbiosis in maize. J. Plant Physiol. 162, 1210–1219. doi: 10.1016/j.jplph.2005.01.014 16323272

[B43] Floss D. S. Gomez S. K. Park H. J. MacLean A. M. Müller L. M. Bhattarai K. K. . (2017). A transcriptional program for arbuscule degeneration during AM symbiosis is regulated by MYB1. Curr. Biol. 27, 1206–1212. doi: 10.1016/j.cub.2017.03.003 28392110

[B44] Floss D. S. Levy J. G. Lévesque-Tremblay V. Pumplin N. Harrison M. J. (2013). DELLA proteins regulate arbuscule formation in arbuscular mycorrhizal symbiosis. Proc. Natl. Acad. Sci. U.S.A. 110, E5025-34. doi: 10.1073/pnas.1308973110 24297892 PMC3870710

[B45] Foo E. Ross J. J. Jones W. T. Reid J. B. (2013). Plant hormones in arbuscular mycorrhizal symbioses: An emerging role for gibberellins. Ann. Bot. 111, 769–779. doi: 10.1093/aob/mct041 23508650 PMC3631329

[B46] Frenzel A. Manthey K. Perlick A. M. Meyer F. Pühler A. Küster H. . (2005). Combined transcriptome profiling reveals a novel family of arbuscular mycorrhizal-specific Medicago truncatula lectin genes. Mol. Plant-Microbe Interact. 18, 771–782. doi: 10.1094/mpmi-18-0771 16134889

[B47] Frenzel A. Tiller N. Hause B. Krajinski F. (2006). The conserved arbuscular mycorrhiza-specific transcription of the secretory lectin MtLec5 is mediated by a short upstream sequence containing specific protein binding sites. Planta 224, 792–800. doi: 10.1007/s00425-006-0262-8 16596411

[B48] Ge S. Yuan K. Lei M. (2026). The SPX protein family in plants: From phosphate sensors to multifunctional signaling hubs. Stress Biol. 6, 36. doi: 10.1007/s44154-026-00307-3 42098336 PMC13153306

[B49] Genre A. Russo G. (2016). Does a common pathway transduce symbiotic signals in plant-microbe interactions? Front. Plant Sci. 7, 96. doi: 10.3389/fpls.2016.00096 26909085 PMC4754458

[B50] Glassop D. Smith S. E. Smith F. W. (2005). Cereal phosphate transporters associated with the mycorrhizal pathway of phosphate uptake into roots. Planta. 222, 688–698. doi: 10.1007/s00425-005-0015-0 16133217

[B51] Gobbato E. Marsh J. F. Vernié T. Wang E. Maillet F. Kim J. . (2012). A GRAS-type transcription factor with a specific function in mycorrhizal signaling. Curr. Biol. 22, 2236–2241. doi: 10.1016/j.cub.2012.09.044 23122845

[B52] González E. Solano R. Rubio V. Leyva A. Paz-Ares J. (2005). PHOSPHATE TRANSPORTER TRAFFIC FACILITATOR1 is a plant-specific SEC12-related protein that enables the endoplasmic reticulum exit of a high-affinity phosphate transporter in Arabidopsis. Plant Cell 17, 3500–3512. doi: 10.1105/tpc.105.036640 PMC1315384 16284308

[B53] Großkinsky D. K. Molin E. M. Bosetto F. Edelsbrunner K. Oravec M. Večeřová K. . (2025). Structure-function relation of cytokinins determines their differential efficiency in mediating tobacco resistance against Pseudomonas syringae. Physiol. Plant 177, e70028. doi: 10.1111/ppl.70028 PMC11672182 39727031

[B54] Gu M. Chen A. Dai X. Liu W. Xu G. (2011). How does phosphate status influence the development of the arbuscular mycorrhizal symbiosis? Plant Signal. Behav. 6, 1300–1304. doi: 10.4161/psb.6.9.16365 22019636 PMC3258057

[B55] Guether M. Balestrini R. Hannah M. He J. Udvardi M. K. Bonfante P. (2009). Genome-wide reprogramming of regulatory networks, transport, cell wall and membrane biogenesis during arbuscular mycorrhizal symbiosis in Lotus japonicus. New Phytol. 182, 200–212. doi: 10.1111/j.1469-8137.2008.02725.x 19192192

[B56] Güimil S. Chang H. S. Zhu T. Sesma A. Osbourn A. Roux C. . (2005). Comparative transcriptomics of rice reveals an ancient pattern of response to microbial colonization. Proc. Natl. Acad. Sci. U.S.A. 102, 8066–8070. doi: 10.1073/pnas.0502999102 PMC1142390 15905328

[B57] Guo R. Wu Y. N. Liu C. C. Liu Y. N. Tian L. Cheng J. F. . (2022). OsADK1, a novel kinase regulating arbuscular mycorrhizal symbiosis in rice. New Phytol. 234, 256–268. doi: 10.1111/nph.17979 35133010

[B58] Gutjahr C. Banba M. Croset V. An K. Miyao A. An G. . (2008). Arbuscular mycorrhiza-specific signaling in rice transcends the common symbiosis signaling pathway. Plant Cell 20, 2989–3005. doi: 10.1105/tpc.108.062414 19033527 PMC2613669

[B59] Harrison M. J. (2005). Signaling in the arbuscular mycorrhizal symbiosis. Annu. Rev. Microbiol. 59, 19–42. doi: 10.1146/annurev.micro.58.030603.123749 16153162

[B60] Harrison M. J. Dewbre G. R. Liu J. (2002). A phosphate transporter from Medicago truncatula involved in the acquisition of phosphate released by arbuscular mycorrhizal fungi. Plant Cell 14, 2413–2429. doi: 10.1105/tpc.004861 12368495 PMC151226

[B61] Harrison M. J. Van Buuren M. L. (1995). A phosphate transporter from the mycorrhizal fungus Glomus versiforme. Nature 378, 626–629. doi: 10.1038/378626a0 8524398

[B62] Herrera-Medina M. J. Steinkellner S. Vierheilig H. Ocampo Bote J. A. García Garrido J. M. (2007). Abscisic acid determines arbuscule development and functionality in the tomato arbuscular mycorrhiza. New Phytol. 175, 554–564. doi: 10.1111/j.1469-8137.2007.02107.x 17635230

[B63] Ho-Plágaro T. García-Garrido J. M. (2022). Multifarious and interactive roles of GRAS transcription factors during arbuscular mycorrhiza development. Front. Plant Sci. 13, 836213. doi: 10.3389/fpls.2022.836213 35419017 PMC8996055

[B64] Huang L. M. Jia X. X. Zhang G. L. Shao M. A. (2017). Soil organic phosphorus transformation during ecosystem development: A review. Plant Soil 417, 17–42. doi: 10.1007/s11104-017-3240-y30311153

[B65] Huang S. Ma Z. Hu L. Huang K. Zhang M. Zhang S. . (2021). Involvement of rice transcription factor OsERF19 in response to ABA and salt stress responses. Plant Physiol. Biochem. 167, 22–30. doi: 10.1016/j.plaphy.2021.07.027 34329842

[B66] Huang T. K. Han C. L. Lin S. I. Chen Y. J. Tsai Y. C. Chen Y. R. . (2013). Identification of downstream components of ubiquitin-conjugating enzyme PHOSPHATE2 by quantitative membrane proteomics in Arabidopsis roots. Plant Cell 25, 4049–4060. doi: 10.1105/tpc.113.115998 24122829 PMC3877800

[B67] Huisman R. Hontelez J. Bisseling T. Limpens E. (2020). SNARE complexity in arbuscular mycorrhizal symbiosis. Front. Plant Sci. 11, 354. doi: 10.3389/fpls.2020.00354 32308661 PMC7145992

[B68] Ikeh M. A. Kastora S. L. Day A. M. Herrero-de-Dios C. M. Tarrant E. Waldron K. J. . (2016). Pho4 mediates phosphate acquisition in Candida albicans and is vital for stress resistance and metal homeostasis. Mol. Biol. Cell 27, 2784–2801. doi: 10.1091/mbc.e16-05-0266 27385340 PMC5007097

[B69] Ivanov S. Harrison M. J. (2014). A set of fluorescent protein-based markers expressed from constitutive and arbuscular mycorrhiza-inducible promoters to label organelles, membranes and cytoskeletal elements in Medicago truncatula. Plant J. 80, 1151–1163. doi: 10.1111/tpj.12706 25329881

[B70] Javot H. Penmetsa R. V. Breuillin F. Bhattarai K. K. Noar R. D. Gomez S. K. . (2011). Medicago truncatula mtpt4 mutants reveal a role for nitrogen in the regulation of arbuscule degeneration in arbuscular mycorrhizal symbiosis. Plant J. 68, 954–965. doi: 10.1111/j.1365-313x.2011.04746.x 21848683

[B71] Javot H. Penmetsa R. V. Terzaghi N. Cook D. R. Harrison M. J. (2007a). A Medicago truncatula phosphate transporter indispensable for the arbuscular mycorrhizal symbiosis. Proc. Natl. Acad. Sci. U.S.A. 104, 1720–1725. doi: 10.1073/pnas.0608136104 17242358 PMC1785290

[B72] Javot H. Pumplin N. Harrison M. J. (2007b). Phosphate in the arbuscular mycorrhizal symbiosis: transport properties and regulatory roles. Plant Cell Environ. 30, 310–322. doi: 10.1111/j.1365-3040.2006.01617.x 17263776

[B73] Jia J. Lu Y. Li Y. Li Y. Li L. Zhang H. (2025). Functional characterization of Acer truncatum PHT1 family phosphate transporter genes and their involvement in arbuscular mycorrhizal symbiosis. Physiol. Plant 177, e70346. doi: 10.1111/ppl.70346 40556045

[B74] Jiang F. Zhang L. Zhou J. George T. S. Feng G. (2021). Arbuscular mycorrhizal fungi enhance mineralisation of organic phosphorus by carrying bacteria along their extraradical hyphae. New Phytol. 230, 304–315. doi: 10.1111/nph.17081 33205416

[B75] Jiang Y. Wang W. Xie Q. Liu N. Liu L. Wang D. . (2017). Plants transfer lipids to sustain colonization by mutualistic mycorrhizal and parasitic fungi. Science 356, 1172–1175. doi: 10.1126/science.aam9970 28596307

[B76] Jiang Y. Xie Q. Wang W. Yang J. Zhang X. Yu N. . (2018). Medicago AP2-domain transcription factor WRI5a is a master regulator of lipid biosynthesis and transfer during mycorrhizal symbiosis. Mol. Plant 11, 1344–1359. doi: 10.1016/j.molp.2018.09.006 30292683

[B77] Joner E. J. Van Aarle I. M. Vosatka M. (2000). Phosphatase activity of extra-radical arbuscular mycorrhizal hyphae: a review. Plant Soil 226, 199–210. doi: 10.1023/a:102658220719241886696

[B78] Jung J. Y. Ried M. K. Hothorn M. Poirier Y. (2018). Control of plant phosphate homeostasis by inositol pyrophosphates and the SPX domain. Curr. Opin. Biotechnol. 49, 156–162. doi: 10.1016/j.copbio.2017.08.012 28889038

[B79] Karandashov V. Bucher M. (2005). Symbiotic phosphate transport in arbuscular mycorrhizas. Trends Plant Sci. 10, 22–29. doi: 10.1016/j.tplants.2004.12.003 15642520

[B80] Karandashov V. Nagy R. Wegmüller S. Amrhein N. Bucher M. (2004). Evolutionary conservation of a phosphate transporter in the arbuscular mycorrhizal symbiosis. Proc. Natl. Acad. Sci. U.S.A. 101, 6285–6290. doi: 10.1073/pnas.0306074101 15075387 PMC395961

[B81] Kiers E. T. Duhamel M. Beesetty Y. Mensah J. A. Franken O. Verbruggen E. . (2011). Reciprocal rewards stabilize cooperation in the mycorrhizal symbiosis. Science 333, 880–882. doi: 10.1126/science.1208473 21836016

[B82] Kobae Y. (2019). Dynamic phosphate uptake in arbuscular mycorrhizal roots under field conditions. Front. Environ. Sci. 6, 159. doi: 10.3389/fenvs.2018.00159

[B83] Kobae Y. Hata S. (2010). Dynamics of periarbuscular membranes visualized with a fluorescent phosphate transporter in arbuscular mycorrhizal roots of rice. Plant Cell Physiol. 51, 341–353. doi: 10.1093/pcp/pcq013 20097910

[B84] Kokkoris V. (2026). Mycelial dynamics in arbuscular mycorrhizal fungi. New Phytol. 249, 691–713. doi: 10.1111/nph.70688 41140005 PMC12712441

[B85] Konečný J. Hršelová H. Bukovská P. Hujslová M. Jansa J. (2019). Correlative evidence for co-regulation of phosphorus and carbon exchanges with symbiotic fungus in the arbuscular mycorrhizal Medicago truncatula. PloS One 14, e0224938. doi: 10.1371/journal.pone.0224938 PMC6844471 31710651

[B86] Kryukov A. A. Gorbunova A. O. Kudriashova T. R. Yakhin O. I. Lubyanov A. A. Malikov U. M. . (2021). Sugar transporters of the SWEET family and their role in arbuscular mycorrhiza. Vavilovskii Zhurnal Genet. Selektsii 25, 754–760. doi: 10.18699/vj21.086 34950847 PMC8649747

[B87] Küster H. Vieweg M. F. Manthey K. Baier M. C. Hohnjec N. Perlick A. M. (2007). Identification and expression regulation of symbiotically activated legume genes. Phytochemistry 68, 8–18. doi: 10.1016/j.phytochem.2006.09.029 17081575

[B88] Kyslynska A. S. Nadkernychna O. V. Kopylov Y. P. Tsekhmister H. V. (2023). The relation between mutualistic mycorrhiza and endophytic plant-fungus associations and their effect on host plants. Agric. Sci. Pract. 10, 54–75. doi: 10.15407/agrisp10.01.054

[B89] Lei W. Li Y. Yao X. Qiao K. Wei L. Liu B. . (2020). NAP is involved in GA-mediated chlorophyll degradation and leaf senescence by interacting with DELLAs in Arabidopsis. Plant Cell Rep. 39, 75–87. doi: 10.1007/s00299-019-02474-2 31646371

[B90] Leng J. Wei X. Jin X. Wang L. Fan K. Zou K. . (2023). ARBUSCULAR MYCORRHIZA-INDUCED KINASES AMK8 and AMK24 associate with the receptor-like kinase KINASE3 to regulate arbuscular mycorrhizal symbiosis in Lotus japonicus. Plant Cell 35, 2006–2026. doi: 10.1093/plcell/koad050 36808553 PMC10226568

[B91] Li J. Chen S. Yu B. Li Q. Liu R. Wang Z. . (2025). TIR immune signalling is blocked by phosphorylation to maintain plant growth. Nat. Plants 11, 1193–1204. doi: 10.1038/s41477-025-02012-x 40490532

[B92] Li X. L. George E. Marschner H. (1991). Phosphorus depletion and pH decrease at the root–soil and hyphae–soil interfaces of VA mycorrhizal white clover fertilized with ammonium. New Phytol. 119, 397–404. doi: 10.1111/j.1469-8137.1991.tb00039.x40046247

[B93] Li Y. Wang X. Zhang H. Wang S. Ye X. Shi L. . (2019). Molecular identification of the phosphate transporter family 1 (PHT1) genes and their expression profiles in response to phosphorus deprivation and other abiotic stresses in Brassica napus. PloS One 14, e0220374. doi: 10.1371/journal.pone.0220374 31344115 PMC6657917

[B94] Li Y. Xu Z. Zhang L. Chen W. Feng G. (2024). Dynamics between soil fixation of fertilizer phosphorus and biological phosphorus mobilization determine the phosphorus budgets in agroecosystems. Agric. Ecosyst. Environ. 375, 109174. doi: 10.1016/j.agee.2024.10917442574925

[B95] Liao D. Sun C. Liang H. Wang Y. Bian X. Dong C. . (2022). SlSPX1-SlPHR complexes mediate the suppression of arbuscular mycorrhizal symbiosis by phosphate repletion in tomato. Plant Cell 34, 4045–4065. doi: 10.1093/plcell/koac212 35863053 PMC9516199

[B96] Liao D. Wang S. Cui M. Liu J. Chen A. Xu G. (2018). Phytohormones regulate the development of arbuscular mycorrhizal symbiosis. Int. J. Mol. Sci. 19, 3146. doi: 10.3390/ijms19103146 30322086 PMC6213213

[B97] Lin D. Tian P. Zhu X. Lin Z. Li Z. Zhang Y. . (2025). A PHR transcription factor-directed gene network reveals key regulators of phosphate metabolism and starvation responses in tomato. Plant Cell 37, koaf171. doi: 10.1093/plcell/koaf171 40591741

[B98] Lin S. I. Chiang S. F. Lin W. Y. Chen J. W. Tseng C. Y. Wu P. C. . (2008). Regulatory network of microRNA399 and PHO2 by systemic signaling. Plant Physiol. 147, 732–746. doi: 10.1104/pp.108.116269 18390805 PMC2409027

[B99] Lin W. Y. Huang T. K. Chiou T. J. (2013). NITROGEN LIMITATION ADAPTATION, a target of microRNA827, mediates degradation of plasma membrane-localized phosphate transporters to maintain phosphate homeostasis in Arabidopsis. Plant Cell 25, 4061–4074. doi: 10.1105/tpc.113.116012 24122828 PMC3877804

[B100] Liu C. Y. Guo X. N. Dai F. J. Wu Q. S. (2024a). Mycorrhizal symbiosis enhances P uptake and indole-3-acetic acid accumulation to improve root morphology in different citrus genotypes. Horticulturae. 10, 339. doi: 10.3390/horticulturae1004033930654563

[B101] Liu L. Gao Z. Li H. Yang W. Yang Y. Lin J. . (2024b). Thresholds of nitrogen and phosphorus input substantially alter arbuscular mycorrhizal fungal communities and wheat yield in dryland farmland. J. Agric. Food. Chem. 72, 10236–10246. doi: 10.1021/acs.jafc.4c00073 38647353

[B102] Liu W. Kohlen W. Lillo A. Op den Camp R. Ivanov S. Hartog M. . (2011). Strigolactone biosynthesis in Medicago truncatula and rice requires the symbiotic GRAS-type transcription factors NSP1 and NSP2. Plant Cell 23, 3853–3865. doi: 10.1105/tpc.111.089771 22039214 PMC3229154

[B103] Liu X. Yu J. Wei J. Qin Y. Shi X. Liu K. . (2025). Inoculation with arbuscular mycorrhizal fungi at the seedling stage of potatoes improves phosphorus use efficiency. Front. Sustain. Food. Syst. 9, 1546032. doi: 10.3389/fsufs.2025.1546032

[B104] López-Arredondo D. L. Leyva-González M. A. González-Morales S. I. López-Bucio J. Herrera-Estrella L. (2014). Phosphate nutrition: improving low-phosphate tolerance in crops. Annu. Rev. Plant Biol. 65, 95–123. doi: 10.1146/annurev-arplant-050213-035949 24579991

[B105] Loth-Pereda V. Orsini E. Courty P. E. Lota F. Kohler A. Diss L. . (2011). Structure and expression profile of the phosphate Pht1 transporter gene family in mycorrhizal Populus trichocarpa. Plant Physiol. 156, 2141–2154. doi: 10.1104/pp.111.180646 21705655 PMC3149965

[B106] Luginbuehl L. H. Oldroyd G. E. D. (2017). Understanding the arbuscule at the heart of endomycorrhizal symbioses in plants. Curr. Biol. 27, R952–R963. doi: 10.1016/j.cub.2017.06.042 28898668

[B107] Lv Q. Zhong Y. Wang Y. Wang Z. Zhang L. Shi J. . (2014). SPX4 negatively regulates phosphate signaling and homeostasis through its interaction with PHR2 in rice. Plant Cell 26, 1586–1597. doi: 10.1105/tpc.114.123208 24692424 PMC4036573

[B108] Lynch J. P. (2011). Root phenes for enhanced soil exploration and phosphorus acquisition: tools for future crops. Plant Physiol. 156, 1041–1049. doi: 10.1104/pp.111.175414 21610180 PMC3135935

[B109] Ma Z. Hu L. (2024). WRKY transcription factor responses and tolerance to abiotic stresses in plants. Int. J. Mol. Sci. 25, 6845. doi: 10.3390/ijms25136845 38999954 PMC11241455

[B110] Ma Z. Hu L. Jiang W. (2024). Understanding AP2/ERF transcription factor responses and tolerance to various abiotic stresses in plants: a comprehensive review. Int. J. Mol. Sci. 25, 893. doi: 10.3390/ijms25020893 38255967 PMC10815832

[B111] Ma Z. Hu L. Zhong Y. (2025). Structure, evolution, and roles of MYB transcription factors proteins in secondary metabolite biosynthetic pathways and abiotic stresses responses in plants: a comprehensive review. Front. Plant Sci. 16, 1626844. doi: 10.3389/fpls.2025.1626844 40822724 PMC12350382

[B112] Ma Z. Jin Y. M. Wu T. Hu L. Zhang Y. Jiang W. . (2022). OsDREB2B, an AP2/ERF transcription factor, negatively regulates plant height by conferring GA metabolism in rice. Front. Plant Sci. 13, 1007811. doi: 10.3389/fpls.2022.1007811 36388558 PMC9650310

[B113] Ma Z. Wang Q. Hu L. . (2026). Role of phytohormones and nanomaterials in enhancing plant tolerance to abiotic stress. Front Plant Sci. 17, 1857914. doi: 10.3389/fpls.2026.1857914 PMC13372591 42465600

[B114] MacLean A. M. Bravo A. Harrison M. J. (2017). Plant signaling and metabolic pathways enabling arbuscular mycorrhizal symbiosis. Plant Cell 29, 2319–2335. doi: 10.1105/tpc.17.00555 28855333 PMC5940448

[B115] Maeda D. Ashida K. Iguchi K. Chechetka S. A. Hijikata A. Okusako Y. . (2006). Knockdown of an arbuscular mycorrhiza-inducible phosphate transporter gene of Lotus japonicus suppresses mutualistic symbiosis. Plant Cell Physiol. 47, 807–817. doi: 10.1093/pcp/pcj069 16774930

[B116] Maldonado-Mendoza I. E. Dewbre G. R. Harrison M. J. (2001). A phosphate transporter gene from the extra-radical mycelium of an arbuscular mycorrhizal fungus Glomus intraradices is regulated in response to phosphate in the environment. Mol. Plant-Microbe Interact. 14, 1140–1148. doi: 10.1094/mpmi.2001.14.10.1140 11605953

[B117] Mangalakkadan A. Roychowdhury A. Chennakesavulu K. Kumar R. (2026). Phosphate starvation response 1 (PHR1): a versatile master regulator shaping plant resilience beyond phosphate deprivation. J. Exp. Bot. 77, 2156–2176. doi: 10.1093/jxb/erag002 41508829

[B118] Mbodj D. Diedhiou A. G. Manneh B. Ndiaye C. Laplaze L. Kane A. (2025). AMF inoculation reduces yield losses in rice exposed to alternate wetting and drying and low fertilization. Sci. Rep. 15, 12281. doi: 10.1038/s41598-025-95528-3 40210924 PMC11986142

[B119] McGaley J. Orvošová M. Schneider B. Chiu C. H. Roth R. Bowden S. . (2026). Symbiotic phosphate transporter dynamics in rice expose functional plasticity of the arbuscules. Nat. Commun. 17, 5450. doi: 10.1038/s41467-026-71496-8 42020388 PMC13282384

[B120] Mitra D. Panneerselvam P. Chidambaranathan P. Nayak A. K. Kumar A. Kumar U. . (2026). Arbuscular mycorrhizal fungi modulate variety-specific phosphate transporter gene expression in aerobic rice under phosphorus-limited soil conditions. Plants. 15, 1675. doi: 10.3390/plants15111675 42280712 PMC13258995

[B121] Murtaza G. Ahmed Z. Ullah S. Alotaibi M. S. Ali S. Iqbal R. (2026). Hormone-metabolism crosstalk in plants: an integrated signaling network coordinating growth and stress adaptation. Plant Signal. Behav. 21, 2710517. doi: 10.1080/15592324.2026.2710517 42533527 PMC13432824

[B122] Nagarajan V. K. Jain A. Poling M. D. Lewis A. J. Raghothama K. G. Smith A. P. (2011). Arabidopsis Pht1;5 mobilizes phosphate between source and sink organs and influences the interaction between phosphate homeostasis and ethylene signaling. Plant Physiol. 156, 1149–1163. doi: 10.1104/pp.111.174805 21628630 PMC3135966

[B123] Nagy R. Drissner D. Amrhein N. Jakobsen I. Bucher M. (2009). Mycorrhizal phosphate uptake pathway in tomato is phosphorus-repressible and transcriptionally regulated. New Phytol. 181, 950–959. doi: 10.1111/j.1469-8137.2008.02721.x 19140941

[B124] Nagy R. Karandashov V. Chague V. Kalinkevich K. Tamasloukht M. Xu G. . (2005). The characterization of novel mycorrhiza-specific phosphate transporters from Lycopersicon esculentum and Solanum tuberosum uncovers functional redundancy in symbiotic phosphate transport in solanaceous species. Plant J. 42, 236–250. doi: 10.1111/j.1365-313X.2005.02364.x 15807785

[B125] Nagy R. Vasconcelos M. J. Zhao S. McElver J. Bruce W. Amrhein N. . (2006). Differential regulation of five Pht1 phosphate transporters from maize (Zea mays L.). Plant Biol. (Stuttg) 8, 186–197. doi: 10.1055/s-2005-873052 16547863

[B126] Nouri E. Breuillin-Sessoms F. Feller U. Reinhardt D. (2014). Phosphorus and nitrogen regulate arbuscular mycorrhizal symbiosis in Petunia hybrida. PloS One 9, e90841. doi: 10.1371/journal.pone.0090841 24608923 PMC3946601

[B127] Paries M. Gutjahr C. (2023). The good, the bad, and the phosphate: regulation of beneficial and detrimental plant–microbe interactions by the plant phosphate status. New Phytol. 239, 29–46. doi: 10.1111/nph.18933 37145847

[B128] Paries M. Hobecker K. Hernandez, Luelmo S. Binci F. Guercio A. Usländer A. . (2025). The GRAS protein RAM1 interacts with WRI transcription factors to regulate plant genes required for arbuscule development and function. Proc. Natl. Acad. Sci. U.S.A. 122, e2427021122. doi: 10.1073/pnas.2427021122 40388617 PMC12130850

[B129] Park H. J. Floss D. S. Levesque-Tremblay V. Bravo A. Harrison M. J. (2015). Hyphal branching during arbuscule development requires reduced arbuscular mycorrhiza1. Plant Physiol. 169, 2774–2788. doi: 10.1104/pp.15.01155 26511916 PMC4677905

[B130] Park J. Nguyen K. T. Park E. Jeon J. S. Choi G. (2013). DELLA proteins and their interacting RING finger proteins repress gibberellin responses by binding to the promoters of a subset of gibberellin-responsive genes in Arabidopsis. Plant Cell 25, 927–943. doi: 10.1105/tpc.112.108951 23482857 PMC3634697

[B131] Paszkowski U. Jakovleva L. Boller T. (2006). Maize mutants affected at distinct stages of the arbuscular mycorrhizal symbiosis. Plant J. 47, 165–173. doi: 10.1111/j.1365-313x.2006.02785.x 16762030

[B132] Paszkowski U. Kroken S. Roux C. Briggs S. P. (2002). Rice phosphate transporters include an evolutionarily divergent gene specifically activated in arbuscular mycorrhizal symbiosis. Proc. Natl. Acad. Sci. U.S.A. 99, 13324–13329. doi: 10.1073/pnas.202474599 12271140 PMC130632

[B133] Pimprikar P. Gutjahr C. (2018). Transcriptional regulation of arbuscular mycorrhiza development. Plant Cell Physiol. 59, 673–684. doi: 10.1093/pcp/pcy024 29425360

[B134] Pimprikar P. Carbonnel S. Paries M. Katzer K. Klingl V. Bohmer M. . (2016). A CCaMK-CYCLOPS-DELLA complex activates transcription of RAM1 to regulate arbuscule branching. Curr. Biol. 26, 987–998. doi: 10.1016/j.cub.2016.04.021 27020747

[B135] Puga M. I. Mateos I. Charukesi R. Wang Z. Franco-Zorrilla J. M. de Lorenzo L. . (2014). SPX1 is a phosphate-dependent inhibitor of phosphate starvation response 1 in Arabidopsis. Proc. Natl. Acad. Sci. U.S.A. 111, 14947–14952. doi: 10.1073/pnas.1404654111 25271326 PMC4205628

[B136] Pumplin N. Harrison M. J. (2009). Live-cell imaging reveals periarbuscular membrane domains and organelle location in Medicago truncatula roots during arbuscular mycorrhizal symbiosis. Plant Physiol. 151, 809–819. doi: 10.1104/pp.109.141879 19692536 PMC2754618

[B137] Qi S. Wang J. Wan L. Dai Z. da Silva, Matos D. M. Du D. . (2022). Arbuscular mycorrhizal fungi contribute to phosphorous uptake and allocation strategies of Solidago canadensis in a phosphorous-deficient environment. Front. Plant Sci. 13, 831654. doi: 10.3389/fpls.2022.831654 35401639 PMC8987128

[B138] Rae A. L. Cybinski D. H. Jarmey J. M. Smith F. W. (2003). Characterization of two phosphate transporters from barley; evidence for diverse function and kinetic properties among members of the Pht1 family. Plant Mol. Biol. 53, 27–36. doi: 10.1023/b:plan.0000009259.75314.15 14756304

[B139] Rasmussen S. R. Füchtbauer W. Novero M. Volpe V. Malkov N. Genre A. . (2016). Intraradical colonization by arbuscular mycorrhizal fungi triggers induction of a lipochitooligosaccharide receptor. Sci. Rep. 6, 29733. doi: 10.1038/srep29733 27435342 PMC4951684

[B140] Rausch C. Daram P. Brunner S. Jansa J. Laloi M. Leggewie G. . (2001). A phosphate transporter expressed in arbuscule-containing cells in potato. Nature 414, 462–470. doi: 10.1038/35106601 11719809

[B141] Rey T. Bonhomme M. Chatterjee A. Gavrin A. Toulotte J. Yang W. . (2017). The Medicago truncatula GRAS protein RAD1 supports arbuscular mycorrhiza symbiosis and Phytophthora palmivora susceptibility. J. Exp. Bot. 68, 5871–5881. doi: 10.1093/jxb/erx398 29186498 PMC5854134

[B142] Rodriguez-Garcia D. R. Müller L. M. (2026). Signaling at the interface: The cell wall, peptides, and extracellular vesicles mediate partner communication during arbuscular mycorrhizal symbiosis. Curr. Opin. Plant Biol. 89, 102849. doi: 10.1016/j.pbi.2025.102849 41455409

[B143] Rosling A. Midgley M. G. Cheeke T. Urbina H. Fransson P. Phillips R. P. (2016). Phosphorus cycling in deciduous forest soil differs between stands dominated by ecto- and arbuscular mycorrhizal trees. New Phytol. 209, 1184–1195. doi: 10.1111/nph.13720 26510093

[B144] Ruan W. Guo M. Cai L. Hu H. Li C. Liu Y. . (2015). Genetic manipulation of a high-affinity PHR1 target cis-element to improve phosphorous uptake in Oryza sativa L. Plant Mol. Biol. 87, 429–440. doi: 10.1007/s11103-015-0289-y 25657119

[B145] Rui W. Ma J. Wei N. Zhu X. Li Z. (2023). Genome-wide analysis of the PHT gene family and its response to mycorrhizal symbiosis in tomatoes under phosphate starvation conditions. Int. J. Mol. Sci. 24, 10246. doi: 10.3390/ijms241210246 37373390 PMC10298980

[B146] Rui W. Mao Z. Li Z. (2022). The roles of phosphorus and nitrogen nutrient transporters in the arbuscular mycorrhizal symbiosis. Int. J. Mol. Sci. 23, 11027. doi: 10.3390/ijms231911027 36232323 PMC9570102

[B147] Russo G. Genre A. (2021). Divide and be conquered-cell cycle reactivation in arbuscular mycorrhizal symbiosis. Front. Plant Sci. 12, 753265. doi: 10.3389/fpls.2021.753265 34759945 PMC8573090

[B148] Ryan M. H. Tibbett M. Edmonds-Tibbett T. Suriyagoda L. D. Lambers H. Cawthray G. R. . (2012). Carbon trading for phosphorus gain: the balance between rhizosphere carboxylates and arbuscular mycorrhizal symbiosis in plant phosphorus acquisition. Plant Cell Environ. 35, 2170–2180. doi: 10.1111/j.1365-3040.2012.02547.x 22632405

[B149] Samanta I. Ghosh K. Saikia R. Savita Maity P. J. Chowdhary G. (2025). Arbuscular mycorrhizal fungi - a natural tool to impart abiotic stress tolerance in plants. Plant Signal. Behav. 20, 2525843. doi: 10.1080/15592324.2025.2525843 40630045 PMC12243912

[B150] Sega P. Pacak A. (2019). Plant PHR transcription factors: Put on a map. Genes (Basel) 10, 1018. doi: 10.3390/genes10121018 31817743 PMC6947268

[B151] Serrano K. Bezrutczyk M. Goudeau D. Dao T. O'Malley R. Malmstrom R. R. . (2024). Spatial co-transcriptomics reveals discrete stages of the arbuscular mycorrhizal symbiosis. Nat. Plants 10, 673–688. doi: 10.1038/s41477-024-01666-3 38589485 PMC11035146

[B152] Shi J. Zhao B. Zheng S. Zhang X. Wang X. Dong W. . (2021). A phosphate starvation response-centered network regulates mycorrhizal symbiosis. Cell. 184, 5527–5540. doi: 10.1016/j.cell.2021.09.030 34644527

[B153] Singh S. Parniske M. (2012). Activation of calcium- and calmodulin-dependent protein kinase (CCaMK), the central regulator of plant root endosymbiosis. Curr. Opin. Plant Biol. 15, 444–453. doi: 10.1016/j.pbi.2012.04.002 22727503

[B154] Smith F. A. Smith S. E. (1997). Structural diversity in (vesicular)-arbuscular mycorrhizal symbioses. New Phytol. 137, 373–388. doi: 10.1046/j.1469-8137.1997.00848.x 33863081

[B155] Smith S. E. Jakobsen I. Grønlund M. Smith F. A. (2011). Roles of arbuscular mycorrhizas in plant phosphorus nutrition: interactions between pathways of phosphorus uptake in arbuscular mycorrhizal roots have important implications for understanding and manipulating plant phosphorus acquisition. Plant Physiol. 156, 1050–1057. doi: 10.1104/pp.111.174581 21467213 PMC3135927

[B156] Smith S. E. Smith F. A. (2011). Roles of arbuscular mycorrhizas in plant nutrition and growth: new paradigms from cellular to ecosystem scales. Annu. Rev. Plant Biol. 62, 227–250. doi: 10.1146/annurev-arplant-042110-103846 21391813

[B157] Solaiman M. Z. Ezawa T. Kojima T. Saito M. (1999). Polyphosphates in intraradical and extraradical hyphae of an arbuscular mycorrhizal fungus, Gigaspora margarita. Appl. Environ. Microbiol. 65, 5604–5606. doi: 10.1128/aem.65.12.5604-5606.1999 10584026 PMC91766

[B158] Stracke S. Kistner C. Yoshida S. Mulder L. Sato S. Kaneko T. . (2002). A plant receptor-like kinase required for both bacterial and fungal symbiosis. Nature 417, 959–962. doi: 10.1038/nature00841 12087405

[B159] Sun T. Li M. Shao Y. Yu L. Ma F. (2017). Comprehensive genomic identification and expression analysis of the phosphate transporter (PHT) gene family in apple. Front. Plant Sci. 8, 426. doi: 10.3389/fpls.2017.00426 28424713 PMC5371654

[B160] Thilakarathne A. S. Liu F. Zou Z. (2025). Plant signaling hormones and transcription factors: key regulators of plant responses to growth, development, and stress. Plants (Basel) 14, 1070. doi: 10.3390/plants14071070 40219138 PMC11990802

[B161] Toth R. Miller R. M. (1984). Dynamics of arbuscule development and degeneration in a Zea mays mycorrhiza. Am. J. Bot. 71, 449–460. doi: 10.1002/j.1537-2197.1984.tb12530.x42587375

[B162] Volpe V. Giovannetti M. Sun X. G. Fiorilli V. Bonfante P. (2016). The phosphate transporters LjPT4 and MtPT4 mediate early root responses to phosphate status in nonmycorrhizal roots. Plant Cell Environ. 39, 660–671. doi: 10.1111/pce.12659 26476189

[B163] Walder F. Brulé D. Koegel S. Wiemken A. Boller T. Courty P. E. (2015). Plant phosphorus acquisition in a common mycorrhizal network: regulation of phosphate transporter genes of the Pht1 family in sorghum and flax. New Phytol. 205, 1632–1645. doi: 10.1111/nph.13292 25615409

[B164] Wang W. Wang Y. Xie Q. Shi J. Sun L. He Z. . (2026). AP2-domain transcription factor WRI5a-regulated MtABCB1 promotes arbuscule development in mycorrhizal symbiosis. Sci. Bull. (Beijing) 71, 264–268. doi: 10.1016/j.scib.2025.06.033 40670201

[B165] Wang X. Chi Y. Song S. (2024). Important soil microbiota's effects on plants and soils: a comprehensive 30-year systematic literature review. Front. Microbiol. 15, 1347745. doi: 10.3389/fmicb.2024.1347745 38591030 PMC10999704

[B166] Wang Z. Hu H. Huang H. Duan K. Wu Z. Wu P. (2009). Regulation of OsSPX1 and OsSPX3 on expression of OsSPX domain genes and Pi-starvation signaling in rice. J. Integr. Plant Biol. 51, 663–674. doi: 10.1111/j.1744-7909.2009.00834.x 19566645

[B167] Wang Z. Ruan W. Shi J. Zhang L. Xiang D. Yang C. . (2014). Rice SPX1 and SPX2 inhibit phosphate starvation responses through interacting with PHR2 in a phosphate-dependent manner. Proc. Natl. Acad. Sci. U.S.A. 111, 14953–14958. doi: 10.1073/pnas.1404680111 25271318 PMC4205599

[B168] Wild R. Gerasimaite R. Jung J. Y. Truffault V. Pavlovic I. Schmidt A. . (2016). Control of eukaryotic phosphate homeostasis by inositol polyphosphate sensor domains. Science 352, 986–990. doi: 10.1126/science.aad9858 27080106

[B169] Willmann M. Gerlach N. Buer B. Polatajko A. Nagy R. Koebke E. . (2013). Mycorrhizal phosphate uptake pathway in maize: vital for growth and cob development on nutrient poor agricultural and greenhouse soils. Front. Plant Sci. 4, 533. doi: 10.3389/fpls.2013.00533 24409191 PMC3872827

[B170] Wipf D. Krajinski F. van T. D. Recorbet G. Courty P. E. (2019). Trading on the arbuscular mycorrhiza market: from arbuscules to common mycorrhizal networks. New Phytol. 223, 1127–1142. doi: 10.1111/nph.15775 30843207

[B171] Xie X. Hu W. Fan X. Chen H. Tang M. (2019). Interactions between phosphorus, zinc, and iron homeostasis in nonmycorrhizal and mycorrhizal plants. Front. Plant Sci. 10, 1172. doi: 10.3389/fpls.2019.01172 31616454 PMC6775243

[B172] Xie X. Huang W. Liu F. Tang N. Liu Y. Lin H. . (2013). Functional analysis of the novel mycorrhiza-specific phosphate transporter AsPT1 and PHT1 family from Astragalus sinicus during the arbuscular mycorrhizal symbiosis. New Phytol. 198, 836–852. doi: 10.1111/nph.12188 23442117

[B173] Xie X. Lai W. Che X. Wang S. Ren Y. Hu W. . (2022). A SPX domain-containing phosphate transporter from Rhizophagus irregularis handles phosphate homeostasis at symbiotic interface of arbuscular mycorrhizas. New Phytol. 234, 650–671. doi: 10.1111/nph.17973 35037255

[B174] Xie X. Lin H. Peng X. Xu C. Sun Z. Jiang K. . (2016). Arbuscular mycorrhizal symbiosis requires a phosphate transceptor in the Gigaspora margarita fungal symbiont. Mol. Plant 9, 1583–1608. doi: 10.1016/j.molp.2016.08.011 27688206

[B175] Xu G. H. Chague V. Melamed-Bessudo C. Kapulnik Y. Jain A. Raghothama K. G. . (2007). Functional characterization of LePT4: a phosphate transporter in tomato with mycorrhiza-enhanced expression. J. Exp. Bot. 58, 2491–2501. doi: 10.1093/jxb/erm096 17545228

[B176] Xu Y. Liu F. Rehman S. Dong H. Hu Q. Tu Z. . (2023). Genome-wide analysis of AMT gene family and its response to mycorrhizal symbiosis in maize. J. Plant Growth Regul. 42, 1134–1143. doi: 10.1007/s00344-022-10618-030311153

[B177] Xue L. Cui H. Buer B. Vijayakumar V. Delaux P. M. Junkermann S. . (2015). Network of GRAS transcription factors involved in the control of arbuscule development in Lotus japonicus. Plant Physiol. 167, 854–871. doi: 10.1104/pp.114.255430 25560877 PMC4348782

[B178] Xue L. Klinnawee L. Zhou Y. Saridis G. Vijayakumar V. Brands M. . (2018). AP2 transcription factor CBX1 with a specific function in symbiotic exchange of nutrients in mycorrhizal Lotus japonicus. Proc. Natl. Acad. Sci. U.S.A. 115, E9239–E9246. doi: 10.1073/pnas.1812275115 30209216 PMC6166803

[B179] Yang J. Lan L. Jin Y. Yu N. Wang D. Wang E. (2022). Mechanisms underlying legume–rhizobium symbioses. J. Integr. Plant Biol. 64, 244–267. doi: 10.1111/jipb.13207 34962095

[B180] Yang S. Y. Grønlund M. Jakobsen I. Grotemeyer M. S. Rentsch D. Miyao A. . (2012). Nonredundant regulation of rice arbuscular mycorrhizal symbiosis by two members of the phosphate transporter1 gene family. Plant Cell 24, 4236–4251. doi: 10.1105/tpc.112.104901 23073651 PMC3517247

[B181] Yang S. Y. Lin W. Y. Hsiao Y. M. Chiou T. J. (2024). Milestones in understanding transport, sensing, and signaling of the plant nutrient phosphorus. Plant Cell 36, 1504–1523. doi: 10.1093/plcell/koad326 38163641 PMC11062440

[B182] Yu N. Luo D. Zhang X. Liu J. Wang W. Jin Y. . (2014). A DELLA protein complex controls the arbuscular mycorrhizal symbiosis in plants. Cell Res. 24, 130–133. doi: 10.1038/cr.2013.167 24343576 PMC3879709

[B183] Zeng H. Wang Y. Xu J. Zhang S. Ali S. Qin C. . (2026). Mineral nutrients as regulators of plant flowering time: a molecular perspective. J. Integr. Plant Biol. 68 (8), 2976–99. doi: 10.1111/jipb.70304 42246288 PMC13446643

[B184] Zhang J. Sun J. Chiu C. H. Landry D. Li K. Wen J. . (2024). A receptor required for chitin perception facilitates arbuscular mycorrhizal associations and distinguishes root symbiosis from immunity. Curr. Biol. 34, 1705–1717. doi: 10.1016/j.cub.2024.03.015 38574729 PMC11037463

[B185] Zhang L. Zhou J. George T. S. Limpens E. Feng G. (2022). Arbuscular mycorrhizal fungi conducting the hyphosphere bacterial orchestra. Trends Plant Sci. 27, 402–411. doi: 10.1016/j.tplants.2021.10.008 34782247

[B186] Zhang L. Xu M. Liu Y. Zhang F. Hodge A. Feng G. (2016). Carbon and phosphorus exchange may enable cooperation between an arbuscular mycorrhizal fungus and a phosphate-solubilizing bacterium. New Phytol. 210, 1022–1032. doi: 10.1111/nph.13838 27074400

[B187] Zhang Q. Wang S. Xie Q. Xia Y. Lu L. Wang M. . (2023). Control of arbuscule development by a transcriptional negative feedback loop in Medicago. Nat. Commun. 14, 5743. doi: 10.1038/s41467-023-41493-2 37717076 PMC10505183

[B188] Zhang X. Pumplin N. Ivanov S. Harrison M. J. (2015). EXO70I is required for development of a sub-domain of the periarbuscular membrane during arbuscular mycorrhizal symbiosis. Curr. Biol. 25, 2189–2195. doi: 10.1016/j.cub.2015.06.075 26234213

[B189] Zhao B. Jia X. Yu N. Murray J. D. Yi K. Wang E. (2024). Microbe-dependent and independent nitrogen and phosphate acquisition and regulation in plants. New Phytol. 242, 1507–1522. doi: 10.1111/nph.19263 37715479

[B190] Zhong Y. Chi H. Wu T. Fan W. Su H. Li R. . (2025). Diversity of rhizosphere microbial communities in different rice varieties and their diverse adaptive responses to saline and alkaline stress. Front. Microbiol. 16, 1537846. doi: 10.3389/fmicb.2025.1537846 40264979 PMC12011871

[B191] Zhou J. Hu Q. Xiao X. Yao D. Ge S. Ye J. . (2021). Mechanism of phosphate sensing and signaling revealed by rice SPX1-PHR2 complex structure. Nat. Commun. 12, 7040. doi: 10.2210/pdb7e40/pdb PMC8639918 34857773

[B192] Zhou Y. Fan J. Wu Q. Wang H. Huang X. Liao L. . (2025). Systematic identification of phosphate transporter family 1 (PHT1) genes and their expression profiling in response to low phosphorus and related hormones in Fagopyrum tataricum (L.) Gaertn. Agronomy 15, 576. doi: 10.3390/agronomy1503057630654563

